# Synthesis and Evaluation of Some Uracil Nucleosides as Promising Anti-Herpes Simplex Virus 1 Agents

**DOI:** 10.3390/molecules26102988

**Published:** 2021-05-18

**Authors:** Samir Mohamed Awad, Shima Mahmoud Ali, Yara Essam Mansour, Samar Said Fatahala

**Affiliations:** 1 Department of Pharmaceutical Organic Chemistry, Faculty of Pharmacy, Helwan University, Ain-Helwan, Cairo 11795, Egypt; Samir.Awad@pharm.helwan.edu.eg (S.M.A.); Yara_mansour@pharm.helwan.edu.eg (Y.E.M.); 2 Department of Microbiology and Immunology, Faculty of Pharmacy, Helwan University, Ain-Helwan, Cairo 11795, Egypt; shaima.mn@gmail.com

**Keywords:** uracil, cyclic nucleosides, acyclic nucleosides, antiviral activities

## Abstract

Since herpes simplex virus type 1 (HSV-1) infection is so widespread, several antiviral drugs have been developed to treat it, among which are uracil nucleosides. However, there are major problems with the current medications such as severe side-effects and drug resistance. Here we present some newly synthesized cyclic and acyclic uracil nucleosides that showed very promising activity against HSV-1 compared to acyclovir.

## 1. Introduction

Viruses are the main causes of fatal infectious diseases affecting humans worldwide [1]. One of the major viral human pathogens is the Herpes virus family, which has the potential to cause lifelong latent infection. Life-threatening diseases can result from the primary infections of the herpes viruses and also from their reactivation, especially in immune-compromised patients [2]. Herpesviridae is a large family of DNA viruses consisting of eight members that are grouped according to biological and genomic similarities into three subfamilies (α, β, and γ) [3]. The γ subfamily includes Kaposi’s sarcoma associated with herpes and the Epstein–Barr virus (EBV). The β subfamily includes cytomegalovirus and the human herpesviruses HHV-6 and HHV-7 [4,5]. The α subfamily includes herpes simplex viruses (HSV-1, HSV-2) and the varicella zoster virus (VZV) [3,6,7], which are among the most common human diseases. HSV usually becomes dormant until reactivated under certain conditions, like emotional stress, fever, and immunosuppression [1]. Symptoms of HSV vary from mild vesicular lesions [8], oral and perioral infections, cold sores and keratitis to serious symptoms such as corneal blindness, encephalitis and disseminated neonatal infections [1,2].

**Figure 1:**
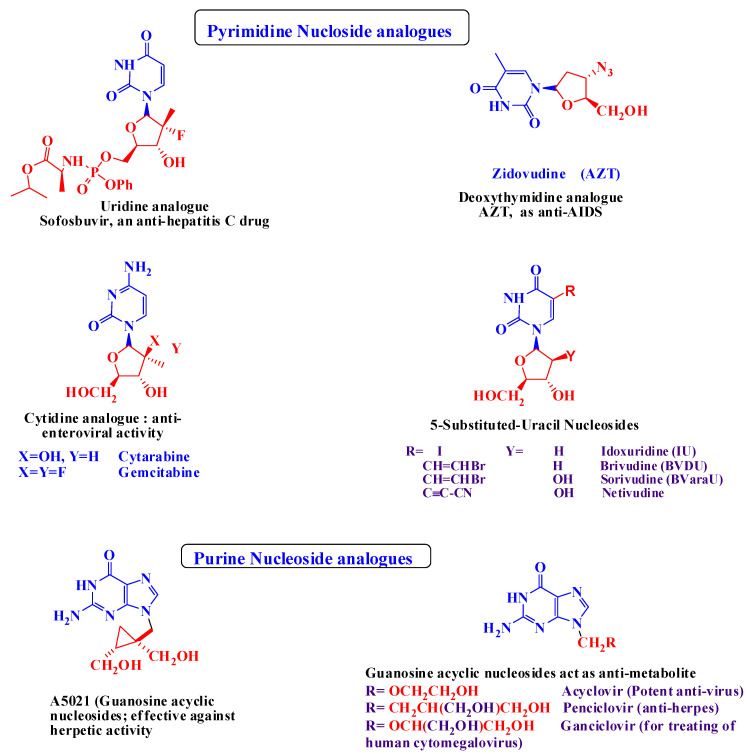
Some potent cyclic and acyclic nucleoside analogues used as potent anti-viral agents [19,20,21,22,23].

The U.S. Food and Drug Administration (FDA) approved antiherpetic drugs belonging to three classes. The first class consists of nucleoside (purine and pyrimidine) analogues in which the sugar moiety is changed [2,8,9]. This class includes acyclovir (ACV) [10] (9-(2-hydroxyethoxymethyl) guanine) [1,2], its valyl ester prodrug valacyclovir (VCV), famciclovir (FCV) (the oral prodrug of penciclovir (PCV)) [11], and ganciclovir (GCV). The second class pertains to acyclic nucleoside phosphonate (ANP) derivatives, and the drug here is cidofovir (CDV). The third class contains pyrophosphate analogues to which belongs phosphonoformic acid or foscarnet (FOS) [7]. Generally speaking, ACV and related drugs are highly successful in treating HSVs [12] because they possess the advantages of metabolic stability, low toxicity and high antiviral potency [2]. However, because of prolonged use, resistant viral strains have emerged, leading to serious clinical problems like severe mucosal infection and visceral dissemination, especially in immunocompromised patients [12]. After their discovery, ANPs, including CDV, became a fundamental class of antiviral drugs for their durable antiviral effects, including on viruses that had become resistant to other drugs [13]. Recently, both CDV and FOS were used to treat severe HSV infections that had become resistant to ACV or FCV [8] but with poor results. The use of CDV and GCV is limited due to poor oral bioavailability and the nephrotoxicity of CDV and hematological toxicity of GCV [2]. Current approaches for improving anti-herpetic activity include a series of 5-substituted 2′-deoxyuridine derivatives such as 5-halovinyl-uracil nucleosides (e.g., brivudin (BVDU, Zostex^®^, Zerpex^®^), and the arabinosyl analogues BVaraU and Sorivudin, which showed particularly potent anti-VZV activity. However, due to the potential toxicity of (BVU), its use as a therapeutic agent is limited [8]. For the abovementioned reasons, research into 5-substituents other than the 5-halovinyl group was encouraged, and for this the newly prepared cyclic sugar moieties have demonstrated specific anti-VZV activity [14,15,16,17,18]. The chemical structures of some of the drugs mentioned above, together with other potent anti-viral agents belonging to the class of cyclic and acyclic nucleoside analogues, are discussed in Figure 1.

**Figure 2:**
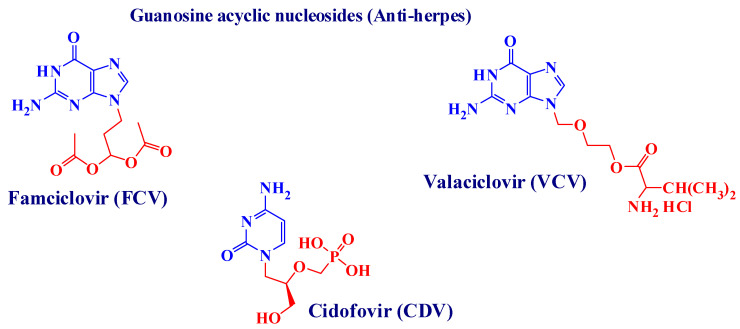
Some anti-viral approved drugs used in clinical trials for the treatment of SARS-CoV-2.

The COVID-19 pandemic has driven the whole world into a rush to invent anti-COVID drugs [24,25]. The RNA-dependent RNA polymerase (RdRp) is essential for coronaviral replication and transcription, and that marks it as the primary target for the antiviral nucleotide analogue drugs [26,27]. Researchers have identified quite a few molecules that interfere with the polymerase reaction, some of which are already FDA-approved to treat other viruses [28,29]. Among these drugs is favipiravir, which has been proven effective in clinical trials to treat SARS-CoV-2 [29,30,31] and has the ability to shut down the polymerase reaction. Our immune system can easily destroy SARS-CoV-2 if it can stop the polymerase reaction [30,32]. The target of all currently available drugs for treating herpes infections is a viral DNA polymerase [33]. Chemically, polymerase inhibitors are classified into two main groups [34,35]: nucleoside analogues and non-nucleoside inhibitors (pyrophosphate derivatives) [36,37]. Among these drugs, cidofovir is an acyclic nucleoside phosphonate approved to treat AIDS and used to treat many other DNA viral infections (e.g., HSV and the papillomavirus) [38,39]. Some of the approved anti-viral drugs are being used in clinical trials to treat SARS-CoV-2, and they are discussed in Figure 2.

The well-known mode of action for nucleoside analogues is through the triphosphate (TP) active form, which allows these analogues to act as competitive inhibitors of the viral DNA polymerase [36]. To become active, the free OH group undergoes three intracellular phosphorylation reaction that convert the nucleoside analogues into (TP) forms. Our newly synthesized compounds are nucleoside analogues that bear the free OH group, which helps them undergo phosphorylation inside the viral cell; however, further investigation into their mode of action must be performed.

Based on the above information, we applied our interest in pyrimidine-derived bioactive molecules to prepare new cyclic and acyclic nucleosides that incorporate 6-substituted-pyrimidine moieties to increase their biological activities.

## 2. Results

### 2.1. Chemical Results

The synthesis of pyrimidine nucleoside analogues was performed via the alkylation of silylated pyrimidine alkylation, according to Vorbrüggen and Niedballa’s procedure [40,41,42]. First, 6-(2,4-dibromophenoxy methyl)-pyrimidine-2,4-dione **1** was prepared via the condensation of ethyl-4-(2,4-dibromophenoxy)-3-oxobutanoate with urea in the presence of sodium ethoxide [43,44,45]. Pyrimidine-2,4-dione **1** on reaction with hexamethyldisilazane (HMDS) [45], afforded Bis(trimethylsilyl) **2**. which was then reacted with different acyclic sugar analogues, namely, 2-acetoxyethyl acetoxymethyl ether **i**, 2-(acetoxymethoxy)propane-1,3-diyldibenzoate **ii** and benzyloxymethyl acetate **iii** to produce the corresponding protected nucleoside analogues **3**, **4**, and **5** respectively. Dialkylation occurred when compound **2** interacted with benzyloxymethyl acetate **iii**, to offer dibenzoxymethyl derivative (**6**) [40,42]. The structures of all the newly prepared compounds were fully characterized by (Mass, ^1^H-NMR and ^13^C-NMR analysis). The ^1^H-NMR showing the disappearance of the NH proton, with the appearance of new signals in the range δ ≈ 5.40–5.45 ppm for O–CH_2_^*^–N, indicated the formation of acyclic analogues **3**, **4** and **5** (^13^C-NMR appearance of oxymethyl C (O–CH_2_^*^–Ph) in the range of 71–72 ppm. For compound **6**, where dialkylation occurred, the disappearance of NH was observed, and two new signals at δ ≈ 5.32 and 5.46 ppm attributed to 2H* of O*CH_2_N^1^ and 2H* of O*CH_2_N^3^ appeared. ^13^C-NMR showed the appearance of the two oxymethyl carbon groups (2* (O–CH_2_^*^–Ph) in the range ≈71–73 ppm)

**Scheme 1:**
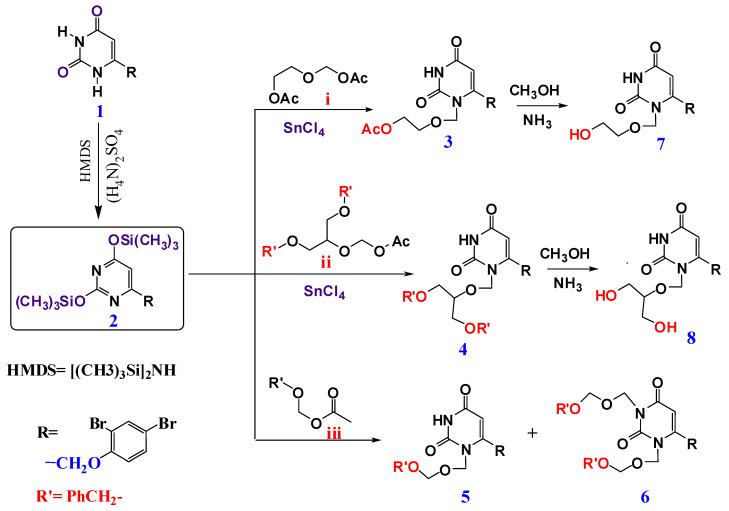
Synthesis of acyclic nucleosides (**3**–**8**).

Protection removal for compounds **3** and **4** was achieved by splitting the ester blocking with an MeOH/NH_3_ solution [46,47,48,49] to give **7** and **8** in a fairly moderate yield (≈55 to 80%), respectively, as revealed in Scheme 1. The ^1^H-NMR showed the appearance of the free OH protons in the range δ ≈ 4.5–4.9 ppm.

**Scheme 2:**
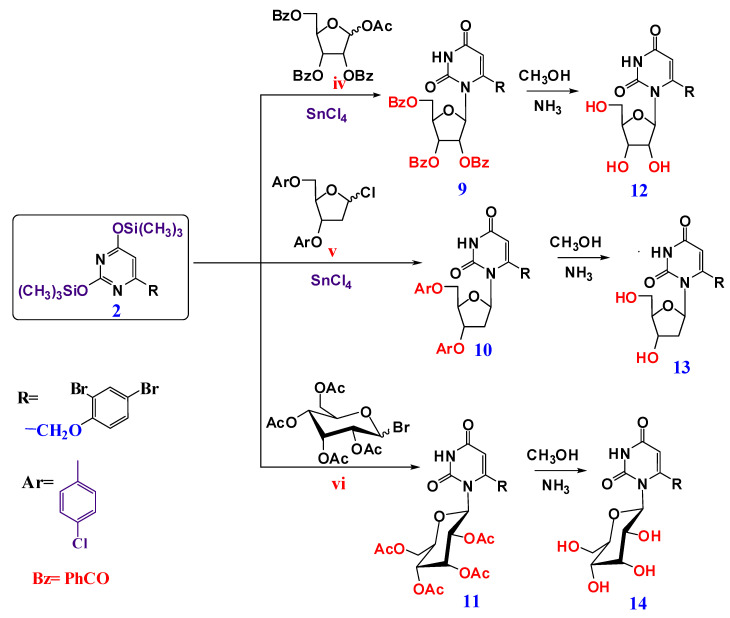
Synthesis of cyclic nucleosides (**9**–**14**).

Cyclic nucleosides (**9–11**) were prepared via the reaction of silyated pyrimidine **2** with various activated cyclic sugars, namely, 1-acetate-2,3,5-tri-0-benzoate-ß-D-ribofuranose **iv**, 2-deoxy-3,5-di-0-p-chlorobenzoyl-D-ribofuranosyl chloride **v** and 1-bromo-2,3,4-tetra-0-acetyl-ß-D-glucopyranose **vi** as reported in [9,50,51], giving the protected nucleosides **9**, **10** and **11** as a ß-anomers. ^1^H-NMR showed a doublet signal in the range δ≈ 6.20–6.48 ppm, corresponding to the anomeric proton of a sugar moiety with a coupling constant (J_1,2_ = 9.10–9.50 Hz) that was attributed to the diaxial orientation of H-1 and H-2 protons, indicating the presence of a ß-configuration. Compounds **9**, **10** and **11** were deprotected by using an MeOH/NH_3_ solution at room temperature [52] to give the compounds **12**, **13**, and **14**, respectively, as revealed in Scheme 2. The ^1^H-NMR showed the appearance of the free OH protons, new signals in the range δ ≈ 3.75–4.6 ppm and the ^13^C-NMR showed the appearance of six carbons from the cyclic sugar moiety in the range ≈62–98 ppm).

### 2.2. Biological Results

#### Antiherpetic Activity of the Synthesized Compounds

Cytopathic effect (CPE) inhibition was evaluated for the 12 synthesized compounds at different concentrations (6, 12, 18, 24, 30, 36, 42, 48, 54, 60, 66, 72, 78 and 84 μg/mL) against the HSV-1 KOS strain. ACV was included as a control in each assay. The percentage values for CPE inhibition were reported in (Table 1).

**Figure 3:**
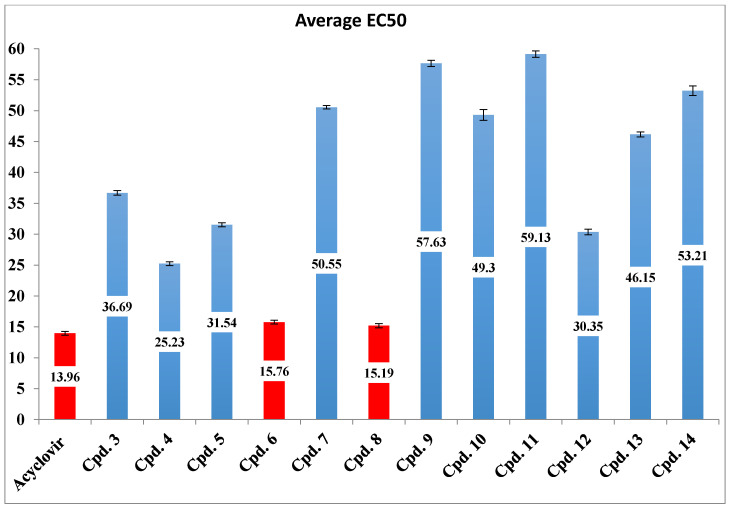
Inhibitory effect of tested compounds and ACV represented by average EC_50_.

From Table 1, we can see that the tested compounds exhibited varied antiherpetic activity compared to that of ACV, yet 6 μg/mL of all compounds and ACV could not prevent CPE presentation. A concentration of 12 μg/mL also could not prevent CPE except for compounds **4**, **6**, and **8**, which gave a CPE inhibition of 14, 30 and 32%, respectively. At a concentration of 36 μg/mL, total prevention of viral CPE presentation was induced in compounds **6** and **8**, while the control drug ACV needed a higher concentration (42 μg/mL) to produce the same effect. The results clearly indicated that two synthetic compounds (**6** and **8**) showed higher antiviral activity than did ACV. Compound **4** also produced total prevention of viral CPE presentation at the same concentration as ACV (42 μg/mL). Antiviral activity was also expressed as the EC_50_, and the results are reported in Figure 3.

According to statistical analysis, it was found that compounds **4**, **6** and **8** were active against HSV-1 with an average EC_50_ of 25.23, 15.76 and 15.1, respectively, which were close to that of ACV (13.96). There was also an insignificant difference (*p* > 0.05) between their EC_50_ values compared to that of ACV, but there was a significant difference (*p* < 0.05) between them and those of other compounds. Some compounds (**3**, **5** and **12**) showed moderate activity l with an EC_50_ of 36.69, 31.54 and 30.35, respectively. The other compounds (**7**, **9**, **10**, **11**, **13** and **14**) showed low activity. The strongest antiviral synthesized compounds were chosen, and the antiviral assay experiment was repeated three times. The results are reported in Table 2.

## 3. Discussion

**Figure 4:**
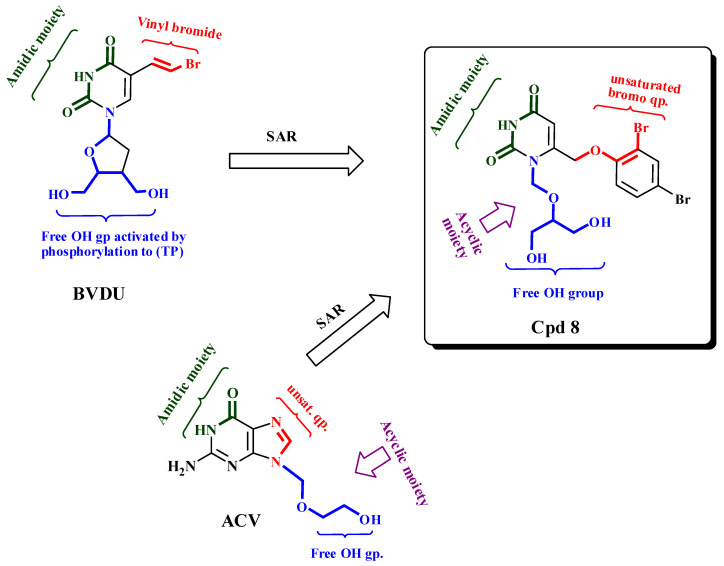
SAR relation between active compounds and reference drug (Acyclovir).

From the abovementioned results, we can conclude that the activity of acyclic nucleosides predominated over that of the cyclic forms. In fact, the acyclics **4**, **6** and **8** showed the highest activity. Compound **4**, the protected form of compound **8** containing *N*-substitution-bearing dibenzyloxymethyl displayed the lowest activity (25.23%), and removing one benzyloxymethyl group from compound **6** increased its activity (15.76%); these two findings indicated that the addition of a bulky group in an acyclic moiety reduced its activity. The highest activity, given by compound **8** (15.19%), occurred after the hydrolysis of a benzoyl group into a free hydroxyl group, indicating the importance of the hydrophilicity the free OH group provided to the activity. Compound **8** was similar to ACV in its free amidic moiety and free-OH acyclic sugar. On the other hand, the cyclic form carrying the *O*-acetyl-*ß-D*-glucopyranosyl moiety, as in compound **11** showed the lowest activity (59.13%) compared to ACV, indicating that the bulkiness of the *N^1^*-substitution decreased activity as shown in Figure 4.

In this study, some of the synthetic compounds, uracil nucleoside analogues, showed very good antiherpetic activity almost equal to that of ACV as shown in Figure 2 and Table 2. Uracil nucleosides are one of the two main groups that inhibits the polymerase [10], which has been studied over many years and represents a highly important target for antiherpetic drugs [53,54]. Another example of uracil nucleoside analogues with a significant antiherpetic activity was presented by Mansour and co-workers (1994). A thiouridine derivative was effective against both HSV-1 and HSV-2 [16,55].

Our results are very promising as these compounds could work to overcome the current resistance to antiherpetic drugs already in use. ACV and related nucleoside analogues have been the gold-standard molecules for treating HSV infections during the past decades [1]; however, the long-term use of antiherpetic drugs has led to selective strain resistance [56,57,58]. These HSV mutants can lead to more severe and chronic infections in immunocompromised patients [1] and even a significant morbidity [56]. For all those reasons, HSV resistance is presented as a major clinical problem for immunocompromised patients [1]. The fact that the numbers of transplant and cancer patients are escalating makes it obvious why the emergence of drug-resistant HSV infections is now a common problem [1]. There are several resistance mechanisms of HSV to ACV ^2^; (**a**) decreased viral TK production, (**b**) complete deficiency of viral TK activity, and (**c**) altered substrate specificity of the viral TK protein and DNA polymerase [7].

Resistance to another nucleoside analogue (VCV, the prodrug of ACV) has also been reported, and the mechanism of resistance is identical to that of ACV [59]. PCV and its prodrug FCV, which are also among the gold-standard agents for the prophylaxis and treatment of HSV-1, have been reported to induce resistance in HSV-1. FOS is an antiviral drug that can overcome HSV-developed ACV resistance [60], but it causes nephrotoxicity, which is a major drawback that limits its clinical use [61,62]. Moreover, some mutants with double resistance to both ACV and FOS can also occur [56]. Another drug that is able to overcome HSV resistance is CDV, an acyclic nucleoside phosphonate, that is reported to be effective against ACV and FOS-resistant HSV in immunocompromised patients [63]. However, it is also nephrotoxic [64], and mutations that have cross-resistance to ACV and FOS have reduced susceptibility to it [65]. 

Because polymerase target binding sites are highly conserved among virus families [10], nucleoside analogue inhibitors possess a relatively high barrier to viral resistance, meaning that the nucleoside analogues presented in our study represent a very good approach for the treatment of drug-resistant HSV-1.

## 4. Materials and Methods

### 4.1. Chemistry

All reagents and solvents were purchased from Merck (Darmstadt, Germany) and used without further purification. All melting points were uncorrected and measured using Electro-thermal IA 9100 apparatus (Shimadzu, Kyoto, Japan). The NMR spectra were recorded on Bruker AMX400 and Bruker Current AV400 Data spectrometer (400 MHz for ^1^H, 100.6 MHz for ^13^C), Bruker BioSpin GmbH, Rheinstetten, Germany. Spectra and chemical shifts (δ) were expressed as ppm against TMS as an internal reference. ESI mass spectra using a Finnigan Thermo Quest MAT 95XL spectrometer and FAB high-resolution (HR) mass spectra using a VG Analytical 70-250S spectrometer (Palmer, Hampden, MA, USA) were conducted using an MCA method with polyethylene glycol as a support. The reactions were monitored by thin layer chromatography (TLC) analysis using silica gel (60 F254)-coated aluminum plates (Merck), which were visualized by UV irradiation (254 nm) and iodine vapors. Column chromatography was performed using 60–120 mesh silica gel. All reactions were carried out under the influence of dry nitrogen.

#### 4.1.1. Preparation of 4-((2,4-Dibromophenoxy)methyl)-2,6-bis (trimethylsilyloxy) pyrimidine **2**

Uracil **1** (3.8 g, 10 mmol), (NH_4_)_2_SO_4_ (10 g, 7.5 mmol) in [(CH_3_)_3_Si]_2_NH) (HMDS) (50 mL, 2.25 mmol) was refluxed with stirring for 4 h. The reaction solvent was evaporated under reduced pressure to give compound **2**.

#### 4.1.2. General Procedure for Preparation of Acyclic and Cyclic Nucleosides

A mixture of (10 mmol) acylated reagents was carried out in dry acetonitrile (30 mL), and these consisted of **i**, acyclic 2-acetoxyethyl acetoxymethyl ether; **ii**, 2-(acetoxymethoxy) propane-1,3-diyldibenzoate; **iii**, benzyloxymethyl acetate; **iv**, cyclic 1-acetate-2,3,5-tri-*O*-benzoate-*ß-D*-ribofuranose; **v**, 2-deoxy-3,5-di-*O*-p-chlorobenzoyl-*D*-ribofuranosyl chloride; and **vi**, 1-bromo-2,3,4-tetra-*O*-acetyl-*ß-D*-glucopyranose. SnCl_4_ (2 mL) was added to the residue of **2** (10 mmol) and stirred at −30 °C for 24 h. The reaction mixture was treated with dry pyridine (4 mL), left until an inorganic residue formed then filtered off. The filtrate was diluted with CHCl_3_ (40 mL), washed with a saturated solution of NaHCO_3_ (50 mL), then a 1 N solution of HCl (50 mL), followed by brine (50 mL) and H_2_O (50 mL) successively. The mixture was dried over anhydrous Na_2_SO_4_ and concentrated until drying under reduced pressure. The residue was separated by silica-gel column chromatography (graduated mixture of CH_3_COOEt and petroleum ether ratio 9:1) to give acyclic nucleosides (**3**–**6**) and cyclic nucleosides (**9**–**11**) (Supplementary Materials).

##### 1-[(2-Acetoxyethoxy)methyl]-6-(2,4-dibromophenoxymethyl)uracil (**3**)

Yield: 1.8 g (70%), m.p. 170–172 °C. ^1^H-NMR (DMSO-d_6_, 400 MHz): δ 2.33 (s,3H, CH_3_C=O) 3.5–4.50 (m, 6H,3*(CH_2_), O-CH_2_), 5.41(s,2H,OCH_2_N^1^), 5.62(s,1H,CH uracil), 7.10–8 (m, 3H,ArH a s,1H,*NH).^13^C-NMR (DMSO-d_6_, 100 MHz): δ 20.5, 63.2, 65.0, 66.7, 72.1, 100.7, 115.9, 116.0, 122.9, 126.1, 128.6, 129.9, 151.5, 151.9, 152.0, 162.7, 170.6. MS (EI) *m/z*: 405.02 [M-AcOCH_2_CH_2_]^+^. Anal. Calcd for C_16_H_16_Br_2_N_2_O_6_: C, 39.05; H, 3.28; N, 5.69; Found C, 39.02; H, 3.31; N, 5.65.

##### 2-[(6-(2,4-dibromophenoxymethyl)-2,4-dioxo-1-pyrimidinyl)methoxy]-1,3-propanediyl dibenzoate (**4**)

Yield: 4.3 g (73%), m.p. 163–165 °C. ^1^H-NMR (DMSO-d_6_, 400 MHz): δ 3.8–4.50 (m, 5H,2*CH_2_,CH), 5.11 (s,2H, CH_2_, phenoxy), 5.43 (s,2H,OCH_2_N), 5.62 (s, 1H, CH uracil), 6.90–8.03 (m, 13H, Ar-H and s,1H,NH8). ^13^C-NMR (DMSO-d_6_, 100 MHz): δ 64.1, 64.8, 71.3, 74.3, 100.6, 115.6, 122.8, 125.9, 128.4, 129.0, 129.5, 129.8, 133.8, 151.3, 151.7, 152.2, 162.5, 165.8. MS (EI) *m/z*: 688.2 [M^+^]. Anal. Calcd for C_29_H_24_Br_2_N_2_O_8_: C, 58.11; H, 4.04; N, 4.67. Found C, 58.24; H, 4.13; N, 4.53.

##### 1-(Benzyloxymethyl)-6-(2,4-dibromophenoxy methyl) uracil (**5**)

Yield: 2.8 g (69%), m.p. 133–135 °C. ^1^H-NMR (DMSO-d_6_, 400 MHz): δ 4.62 (s,2H, CH_2_Ph), 5.25 (s,2H, CH_2_, phenoxy), 5.45 (s,2H,OCH_2_N), 5.81(s, 1H, CH uracil), 7.28–7.81(m, 8H, Ar-H), 8.25 (s,1H,*NH). ^13^C NMR (DMSO-d_6_, 100 MHZ): δ 64.1, 70.3, 71.5, 100.6, 115.6, 122.6, 125.7, 127.0, 127.7, 128.1, 128.2, 129.5, 137.3, 150.9, 151.5, 151.6, 158.8, 162.5, 165.8. MS (EI) *m/z*: 402.2 [M-CH_2_Ph]^+^. Anal. Calcd for C_19_H_16_Br_2_N_2_O_4_: C, 45.99; H, 3.25; N, 5.65. Found C, 45.96: H, 3.29; N, 5.61.

##### 1,3-di(Benzyloxymethyl)-6-(2,4-dibromophenoxy methyl) uracil (**6**)

Yield: 3 g (58%), m.p. 148–150 °C. ^1^H-NMR (DMSO-d_6_, 400 MHz): δ 4.43,4.45 (2s,4H, 2*CH_2_^*^Ph), 5.24 (s,2H, CH_2_, phenoxy), 5.32 (s,2H,OCH_2_N^1^), 5.47 (s,2H,OCH_2_N^3^), 5.81(s, 1H, CH uracil), 7.25–7.67(m, 13H, Ar-H), 8.01(s,1H,*NH). ^13^C NMR (DMSO-d_6_, 100 MHz): δ 65.1, 70.6, 70.9, 71.4, 73.1, 100.2, 116.0, 122.9, 126.1, 127.7, 127.8, 128.1, 128.4, 128.5, 128.6, 129.9, 137.7, 138.8, 150.8, 151.8, 152.2, 161.6. MS (EI) *m/z*: 527.3 [M^+^]. Anal. Calcd for C_27_H_24_Br_2_N_2_O_5_: C, 58.11; H, 4.04; N, 4.67. Found C, 58.24; H, 4.13; N, 4.53.

##### 1-(2,3,5-Tri-O-benzoyl-ß-D-ribofuranosyl)-6-(2,4-dibromophenoxy methyl) uracil (**9**)

Yield: 4.5 g(62%), m.p. 117–119 °C. ^1^H-NMR (DMSO-d_6_, 400 MHz): δ 4.31–4.40 (m, 2H, H-5′,5″) 4.51–4.60 (m, 1H,H-4′), 4.91 (s,2H, CH_2_, phenoxy), 5.71 (s,1H,CH uracil), 5.90–6.11 (m, 2H, H-2′,H-3′), 6.4(d, 1H,j = 9.10 Hz, H-1′), 7.10–7.98(m, 18H, Ar-H), 8.21(s,1H, *NH). ^13^C-NMR (DMSO-d_6_, 100 MHz): δ 63.7, 66.2, 70.7, 73.8, 74.1, 74.5, 78.2, 98.0, 102.9, 115.7, 116.2, 123.0, 125.9, 128.6, 128.8, 128.9, 129.1, 129.2, 129.5, 129.6, 129.7, 129.9, 133.7, 134.0, 134.1, 150.2, 150.9, 151.8, 162.5, 164.9, 165.1, 165.8. MS (EI) *m/z*: 730.2 [M^+^]. Anal. Calcd for C_37_H_28_Br_2_N_2_O_10_: C, 58.11; H, 4.04; N, 4.67. Found C, 58.24; H, 4.13; N, 4.53.

##### ((*2R,3S,5R*)-3-(4-chlorobenzoloxy)-5-(6-((2,4-dibromophenoxy)methyl)-2,4-dioxo-3,4-dihydropyrimidin-1-(*2H*)-yl)tetrahydrofuran-2-yl)methyl-4-chlorobenzoate (**10**)

Yield: 4.5 g (62%), m.p. 117–119 °C. ^1^H-NMR (DMSO-d_6_, 400 MHz): δ 4.31–4.40 (m, 2H,H-5′,5″) 4.51–4.60 (m, 1H,H-4′), 4.91 (s,2H, CH_2_, phenoxy), 5.71 (s,1H,CH uracil), 5.90–6.11 (m, 2H,H-2’,H-3′), 6.4(d, 1H,j = 9.10 Hz, H-1’), 7.10–7.98 (m, 18H, Ar-H), 10.91(br s,1H,NH). ^13^C NMR (DMSO-d_6_, 100 MHZ): δ 34.8, 66.4, 67.6, 74.9, 75.6, 80.3, 82.3, 102.9, 115.7, 128.5, 129.1, 129.2, 129.3, 129.4, 129.8, 129.9, 130.4, 130.6, 131.0, 131.1, 131.3, 131.4, 131.5, 138.6, 150.4, 150.8, 151.1, 162.8, 163.0, 164.9, 165.0. MS (EI) *m/z*: 730.2 [M^+^]. Anal. Calcd for C_37_H_28_Br_2_N_2_O_10_: C, 58.11; H, 4.04; N, 4.67. Found C, 58.24; H, 4.13; N, 4.53.

##### 1-(2,3,4,6-Tetra-*O*-acetyl-*ß-D*-glucopyranosyl)-6-(2,4-dibromophenoxymethyl) uracil (**11**)

Yield: 4.3 g (63%), m.p. 140–142 °C. ^1^H-NMR (DMSO-d_6_, 400 MHz): δ 1.82–2.12 (4s, 12H, 4COCH_3_), 3.32 (m, 1H, H-2′), 5.62(s,1H, CH uracil), 6.21 (d,1H,J1,2 = 9.51 Hz, H-1′), 7.10–7.82 (m, 3H, Ar-H), 10.92 (br s, 1H,NH). ^13^C-NMR (DMSO-d_6_, 100 MHz): δ 15.4, 20.1, 20.4, 20.6, 62.0, 65.2, 65.5, 67.8, 68.0, 68.2, 73.0, 77.7, 79.5, 96.6, 99.2, 115.6, 122.8, 125.8, 149.7, 151.6, 152.2, 162.2, 162.6, 169.2, 170.1. MS (EI) *m/z*: 706.2 [M^+^]. Anal. Calcd for C_25_H_26_Br_2_N_2_O_12_: C, 42.51; H, 3.71; N, 3.97. Found C, 42.64; H, 3.63; N, 3.83.

#### 4.1.3. General Procedure for De-Protection of Nucleosides to Prepare (**7**, **8**, **12**–**14**)

Protected nucleosides (**3**–**5**, **9**–**11**) (10 mmol) were dissolved, individually, in MeOH (20 mL) with NH_3_ (3 mL) and stirred for 48 h at room temperature. The solution was then concentrated to drying, under reduced pressure, and the resulting residue from MeOH was recrystallized to give unprotected nucleosides (**7**, **8**, **12**–**14**).

##### 1-(2-Hydroxyethoxy methyl)-6-(2,4-dibromophenoxy methyl) uracil (**7**)

Yield: 2.8 g (81%), m.p. 222–224 °C. ^1^H-NMR (DMSO-d_6_, 400 MHz): δ 3.71, 3.77 (2t,4H,HOCH_2_^*^CH_2_^*^O), 4.91 (s, 1H,OH*),5.52 (s,2H, Ph-CH_2_^*^), 5.54 (s,2H,OCH_2_N), 6.14 (s, 1H,C-5-H uracil), 7.41–7.81 (m, 3H,Ar-H), 8.11 (s, 1H,NH). ^13^C-NMR (DMSO-d_6_, 100 MHz): δ 60.3, 60.5, 70.2, 80.8, 100.5, 116.0, 122.9, 126.0, 128.6, 129.9, 151.7, 151.1, 152.8, 162.7. MS (EI) *m/z*: 450.07 [M^+^]. Anal. Calcd for C_14_H_14_Br_2_N_2_O_5_: C, 37.36; H, 3.14; N, 6.22. Found C,37.38; H,3.26; N, 6.19.

##### 1-[2-Hydroxy-1-(hydroxyl methyl) ethoxymethyl]-6-(2,4-dibromo phenoxymethyl) uracil (**8**)

Yield: 3.3 g (83%), m.p. 188–190 °C. ^1^H-NMR (DMSO-d_6_, 400 MHz): δ 3.41–3.57 (m, 5H,2* CH_2_,CH), 4.85 (m, 2H, OH*), 5.51 (s, 2H,CH_2_ phenoxy), 5.61(s, 2H, OCH_2_N), 5.87 (s, 1H,CH uracil), 7.31–7.65 (m, 3H,Ar-H), 8.45 (br s,1H,NH*). ^13^C-NMR (DMSO-d_6_, 100 MHz): δ 61.0, 61.4, 70.2, 71.6, 80.5, 100.1, 115.4, 116.4, 122.8, 125.9, 129.6, 129.8, 152.1, 152.2, 163.2. MS (EI) *m/z*: 480.10. Anal. Calcd for C_15_H_16_Br_2_N_2_O_6_: C, 37.53; H, 3.36; N, 5.83. Found C, 37.48; H, 3.39; N, 5.89.

##### 1-(ß-D-Ribofuranosyl)-6-(2,4-dibromophenoxy methyl) uracil (**12**)

Yield: 2.8 g (67%), m.p. 133–135 °C. ^1^H-NMR (DMSO-d_6_, 400 MHz): δ 3.41–3.45 (m, 2H, H-5’,5”), 3.57–3.61(m, 1H,H-4’), 3.71–3.86 (m, 1H,H-3’), 4.10–4.23 (M,1H,H-2’), 4.15 (d,1H,OH*), 4.54(d, 1H,OH*), 4.60 (d,1H,OH), 4.9 (s, 2H,CH_2_ phenoxy), 5.13 (m, 1H,OH), 5.72(s,1H,CH uracil), 6.08(d,1H,7.75 Hz,H-1’),7.20–7.72 (m, 3H,Ar-H), 11.92 (br s, 1H,NH). ^13^C-NMR (DMSO-d_6_,100 MHz): δ 62.6, 65.7, 70.5, 71.2, 84.6, 87.7, 98.1, 115.6, 122.8, 125.8, 128.6, 129.9, 150.4, 152.2, 163.0. MS (EI) *m/z*: 508.11 (M^+^, 15.4%), 510.12(M+2, 14.46%). Anal. Calcd for C_16_H_16_Br_2_N_2_O_7_: C, 37.82; H, 3.17; N, 5.51. Found C, 37.78; H, 3.29; N, 5.60.

##### 6-(2,4-Dibromophenoxy)methyl)-1-((2R,4S,5R)-4-hydroxy-5-(hydroxylmethyl)tetra-hydrofuran-2-yl)pyrimidine-2,4-(*1H,3H*)-dione (**13**)

Yield: 2.6 g (70%), m.p. 186–188 °C. ^1^H-NMR (DMSO-d_6_, 400 MHz): δ 2.24–2.26 (m, 2H, H-2’, 2’’),3.14–3.36 (m, 2H, H-5’,5’’), 3.75 (br s, 2H, 2* OH), 4.53 (m, 1H,H-3’), 4.74 (s, 2H, CH_2_ phenoxy), 5.11 (m, 1H, 4-H’), 5,34 (s, 1H, CH uracil), 5.57 (m, 1H, H-1’), 7.08–7.71 (m, 3H, Ar-H), 10.92 (br s, 1H, NH). ^13^C-NMR (DMSO-d_6_, 100 MHz): δ 61.7, 62.6, 65.7, 70.9, 71.5, 81.4, 86.0, 87.7, 98.1, 115.6, 122.9, 125.8, 128.6, 129.9, 150.7, 151.1, 152.2, 163.2. MS (EI) *m/z*: 492.11(M^+^, 12.8%), 494.11(M+2, 11.7%). Anal. Calcd for C_16_H_16_Br_2_N_2_O_6_: C, 39.05; H, 3.28; N, 5.69. Found C, 39.08; H, 3.27; N, 5.63.

##### 1-(ß-D-Glucopyranosyl)-6-(2,4-dibromophenoxy methyl) uracil (**14**)

Yield: 2.8 g (60.5%), m.p. 205–207 °C. ^1^H-NMR (DMSO-d_6_, 400 MHz): δ 2.82–2.94 (m, 1H, 3H-3’), 3.18–3.48 (m, 4H, H-4’, H-5’, H-6’, 6’’), 4.12 (dd, 1H, J_1,2_ = 9.96, J_2,3_ = 9.16 Hz, H-2’), 4.14 (m, 4H, 4OHs), 4.72 (s, 2H, CH_2_ phenoxy), 5.33 (d, 1H, J = 9.96 Hz, H-1’), 5.55 (s, 1H, CH uracil), 7.04–7.68 (m, 3H, Ar-H), 8.12(br s, 1H, NH*). ^13^C-NMR (DMSO-d_6_,100 MHz): δ 61.1, 65.2, 68.4, 70.9, 78.7, 81.6, 83.4, 96.9, 99.2, 115.8, 122.7, 125.7, 128.6, 129.6, 152.3, 163.8, 166.6. MS (EI) *m/z*: 538.14(M^+^, 9.8%), 540.14 (M+2, 8.7%). Anal. Calcd for C_17_H_18_Br_2_N_2_O_8_: C, 37.94; H, 3.37; N, 5.21. Found C, 37.88; H, 3.39; N, 5.35.

### 4.2. Biology

#### 4.2.1. Cell and Virus

HSV-1 propagation was carried out using the African green monkey kidney cell line (Vero). To culture the cells Dulbeco minimum (Gibco, Paisley, UK) with 10% fetal bovine serum (Gibco) was used. An HSV-1 KOS strain was used examine antiviral activity. Vero cells were used to propagate the virus and the propagated viral stock titer stock was then fixed as TCID 50 mL^−1^ by using Karber’s method. Finally, after the titration, the viral stock was dispensed in sterile tubes, which were then stored at −70 °C for later use [66].

#### 4.2.2. Preparation of Tested Compounds and the Standard

ACV was purchased from Sigma (St. Louis, MO, USA), and the tested compounds were prepared as previously described. Dimethyl sulfoxide (DMSO) was used as a solvent for the tested compounds and ACV.

#### 4.2.3. Antiherpetic Activity Assay

A CPE inhibition assay was used to determine the semi-quantitative antiviral activity of the 12 tested compounds according to [66]. In most studies, DMSO showed an antiviral effect in vitro on different cell types [67]; thus, the concentration of DMSO should be less than 25, which is the lowest allowed concentration for an antiviral effect. Therefore, we ignored its effect in our study when it was used as a solvent for the targeted compounds. The degree of inhibition was expressed as a throughput percentage of virus control (% virus control = CPE experimental group/CPE virus control × 100) [66]. The antiviral activity was also expressed as the EC50, which is the concentration required to reduce a virus-induced CPE or viral plaque formation by 50% compared to the untreated control [2].

## 5. Conclusions

In this research, we presented some new synthetic nucleoside analogues having good activity against HSV-1 equal to or higher than the standard drug, ACV. The acyclic nucleosides predominated over the cyclic forms, with acyclic compounds **6** and **8** giving the highest activities). They induced total prevention of viral CPE presentation at a concentration of 36 μg/mL, which was less than that needed by ACV (42 μg/mL). The EC_50_ values of the compounds were (15.76 and 15.19, respectively, which was close to that of ACV (13.96). Such compounds could be used as alternatives to the currently used antiherpetic drugs, which have major disadvantages such as acquired viral resistance that has become life threatening, especially for immunocompromised patients. With the fact that some antiviral nucleoside analogues are actually being used in some clinical trials to treat SARS-CoV-2, our nucleoside analogues could also serve as promising anti-COVID agents, but of course that needs further study.

## Figures and Tables

**Table 1:** Inhibition of HSV-1-related CPE using different concentrations of tested compounds and a standard drug (ACV).

Concentration (μg/mL)	CPE Inhibition (%)												
	ACV *	Cpd. 3	Cpd. 4 *	Cpd. 5	Cpd. 6 *	Cpd. 7	Cpd. 8 *	Cpd. 9	Cpd. 10	Cpd. 11	Cpd. 12 *	Cpd. 13	Cpd. 14
6	0	0	0	0	0	0	0	0	0	0	0	0	0
12	29	0	14	0	30	0	32	0	0	0	0	0	0
18	50	12	22	15	45	0	42	0	0	0	0	0	0
24	70	28	42	28	65	10	72	0	10	0	32	0	0
30	77	34	62	40	80	16	80	22	20	18	44	22	18
36	92	38	70	65	100	20	100	28	32	22	75	34	28
42	100	55	100	82		26		34	50	26	86	45	36
48		77		100		66		38	82	34	100	52	46
54		80				74		47	100	46		66	58
60		100				88		65		48		72	76
66						100		88		74		77	82
72								100		78		82	100
78										85		100	
84										100			

* Most potent compounds.

**Table 2:** Inhibition of HSV-1-related CPE by using different concentrations of the most potent tested compounds and a standard drug (ACV).

Concentration (μg/mL)	Compound 4	Compound 6	Compound 8	ACV
	CPE Inhibition (%) Mean ± SE			
6	0.00 ± 0.00	0.00 ± 0.00	0.00 ± 0.00	0.00 ± 0.00
12	14.25 ± 3.75	30.30 ± 3.40	31.95 ± 2.75	29.00 ± 3.00
18	22.35 ± 2.25	45.90 ± 3.40	41.25 ± 4.05	49.80 ± 3.40
24	42.35 ± 1.85	65.15 ± 1.75	72.25 ± 2.05	70.45 ± 2.75
32	62.00 ± 5.00	80.40 ± 10.00	80.00 ± 10.00	77.65 ± 1.75
36	75.75 ± 9.85	99.00 ± 1.00	99.00 ± 1.00	92.50 ± 4.50
42	99.50 ± 0.50			99.50 ± 0.50

## Data Availability

The data presented in this study are available on request from the corresponding author.

## Supplementary Materials

The following are available online at. ^1^HNMR and ^13^C NMRspectra copies.
