# Supplementary material for: Synthesis and Evaluation of Some Uracil Nucleosides as Promising Anti-Herpes Simplex Virus 1 Agents
Source: Molecules. 2021 May 18;26(10):2988. doi: 10.3390/molecules26102988 (PMC8157375; doi:10.3390/molecules26102988)
Supplement: Supplementary file 1 [file molecules-26-02988-s001.zip › molecules-1218341-supplementary.pdf]

Supplementary Material

Synthesis and evaluation of some Uracil nucleosides As Promising Anti- Herpes simplex virus 1 Agents

Table of Contents

^{1}H NMR and ^{13}C NMR spectra copies

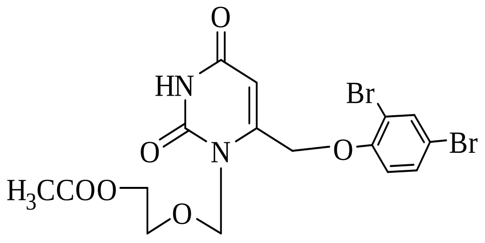

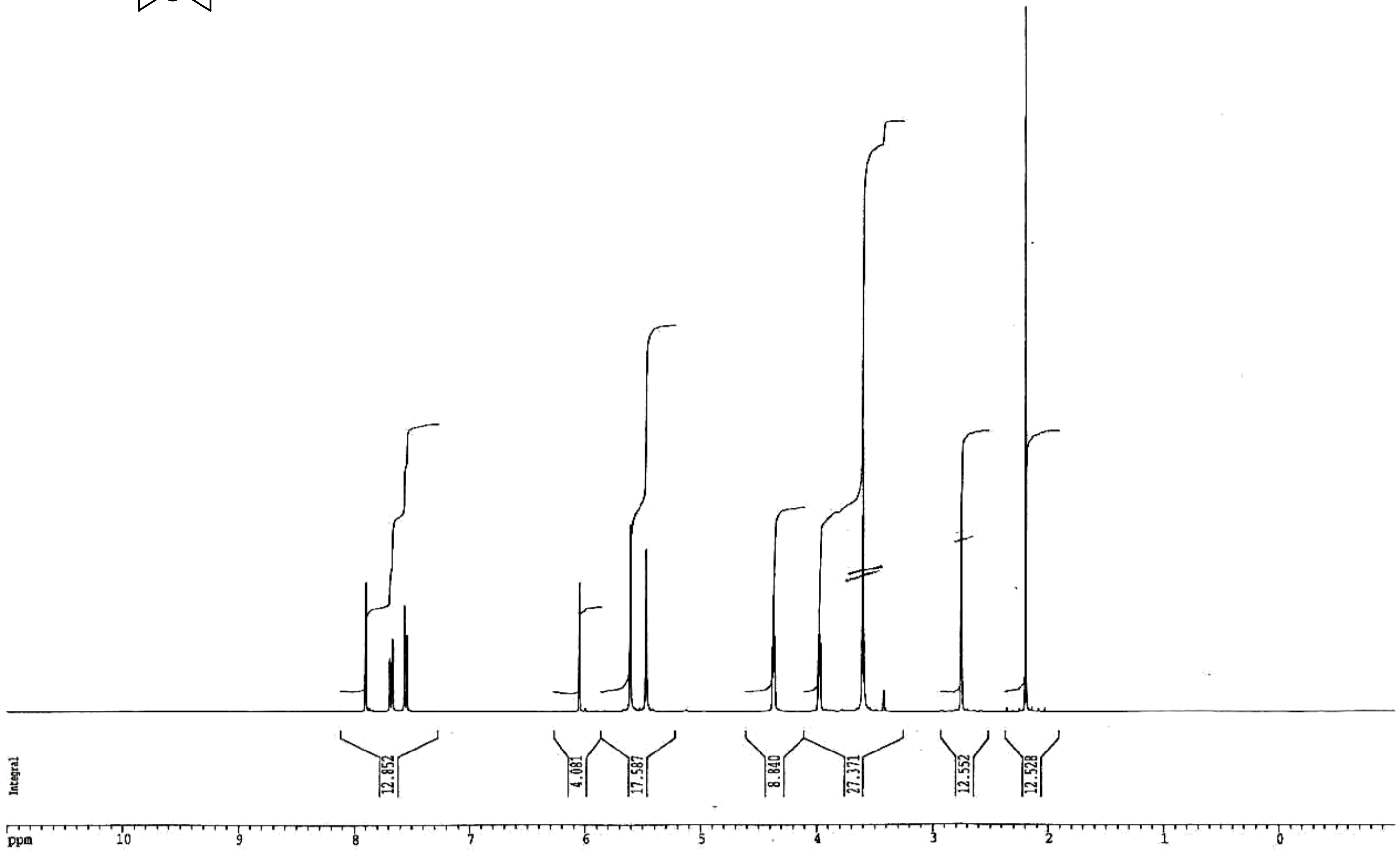

Current AMX400 Data

DU	/x	
NAME	20070511	
EXPNO	310	
PROCNO	1	

F2 - Acquisition Parameters

Date_	20070511	
Time	16.05	
INSTRUM	spect	
PROBHD	5 mm Dual 13	
PULPROG	zg30	
TD	65536	
SOLVENT	DMSO	
NS	16	
DS	2	
SWH	8333.373	Hz
FIDRES	0.127157	Hz
AQ	3.9322100	sec
RG	256	
DW	60.000	usec
DE	85.71	usec
TE	300.0	K
HL1	1	dB
D1	1.00000000	sec
P1	12.50	usec
SFO1	400.1387237	MHz
NUCLEUS	1H	

F2 - Processing parameters

SI	32768	
SF	400.1361882	MHz
WDW	EM	
SSB	0	
LB	0.30	Hz
GB	0	
PC	1.00	

1D NMR plot parameters

CX	30.00	cm
F1P	11.000	ppm
F1	4401.50	Hz
F2P	-1.000	ppm
F2	-400.14	Hz
PPMCM	0.40000	ppm/cm
HZCM	160.05447	Hz/cm

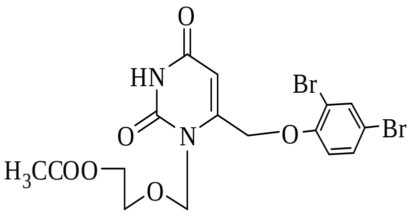

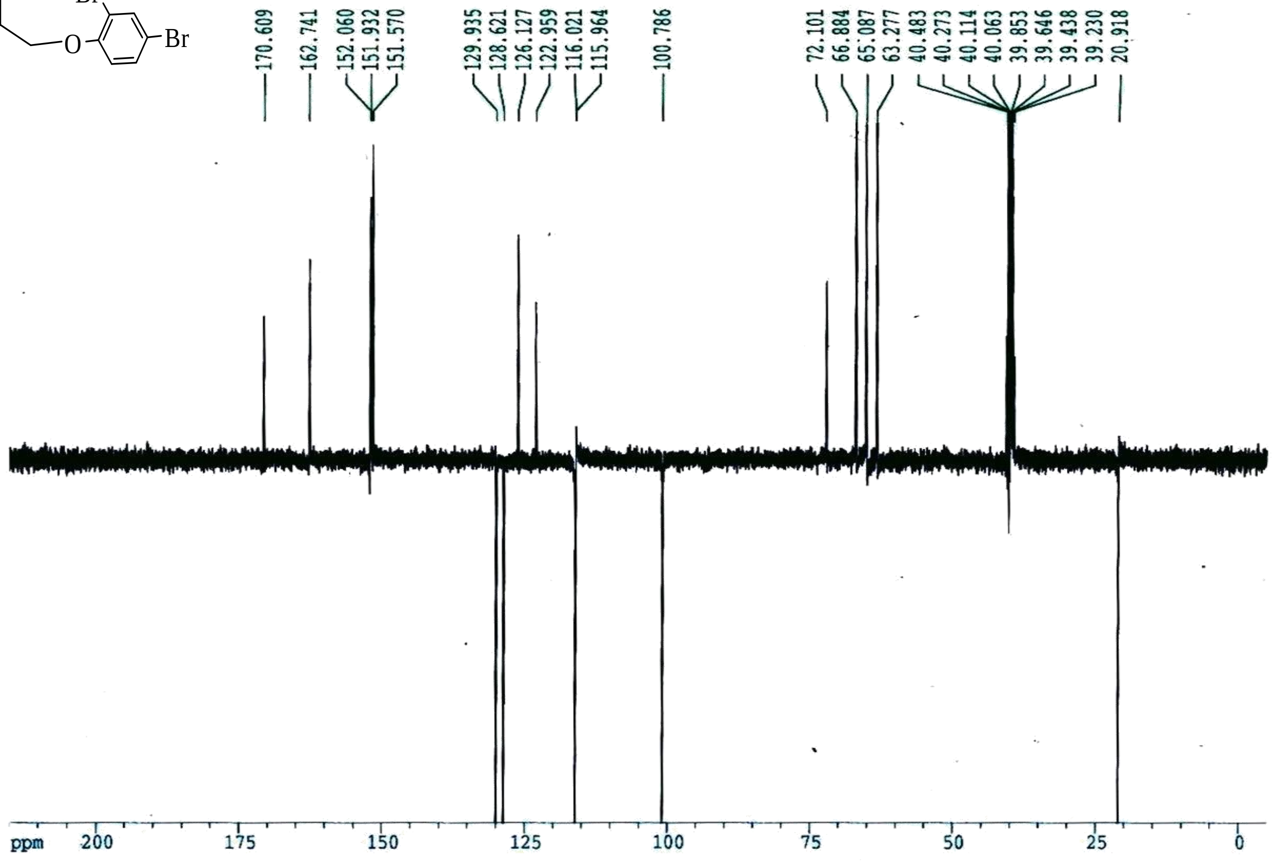


```
Current AMX400 Data
DU                          /x
NAME                  20070514
EXPNO                      270
PROCNO                       1

F2 - Acquisition Parameters
Date_                 20070515
Time                      0.46
INSTRUM                  spect
PROBHD           5 mm Dual 13
PULPROG           pendant.amx
TD                       65536
SOLVENT                   DMSO
NS                         464
DS                           4
SWH               25000.119 Hz
FIDRES             0.381472 Hz
AQ               1.3107700 sec
RG                       32768
DW                20.000 usec
DE                 28.57 usec
TE                   300.0 K
P1                  6.50 usec
P3                 10.60 usec
CNST2          145.0000000
HL1                       1 dB
D1              1.50000000 sec
S1                        1 dB
SFO2          400.1379006 MHz
DECNUC                    1H
D4               0.0017241 sec
P4                  21.2 usec
P2                  13.0 usec
D15              0.0043103 sec
D13              0.0000040 sec
S2                       26 dB
SFO1          100.6245885 MHz
NUCLEUS                   13C
CPDPRG                waltz16
P31                 90.00 usec
D2              0.00357143 sec

F2 - Processing parameters
SI                       32768
SF            100.6143589 MHz
WDW                         EM
SSB                          0
LB                     1.00 Hz
GB                           0
PC                        1.40

1D NMR plot parameters
CX                    20.00 cm
F1P                215.000 ppm
F1                21632.09 Hz
F2P                 -5.000 ppm
F2                 -503.07 Hz
PPMCM          11.00000 ppm/cm
HZCM         1106.75793 Hz/cm
```

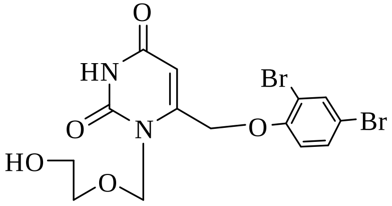

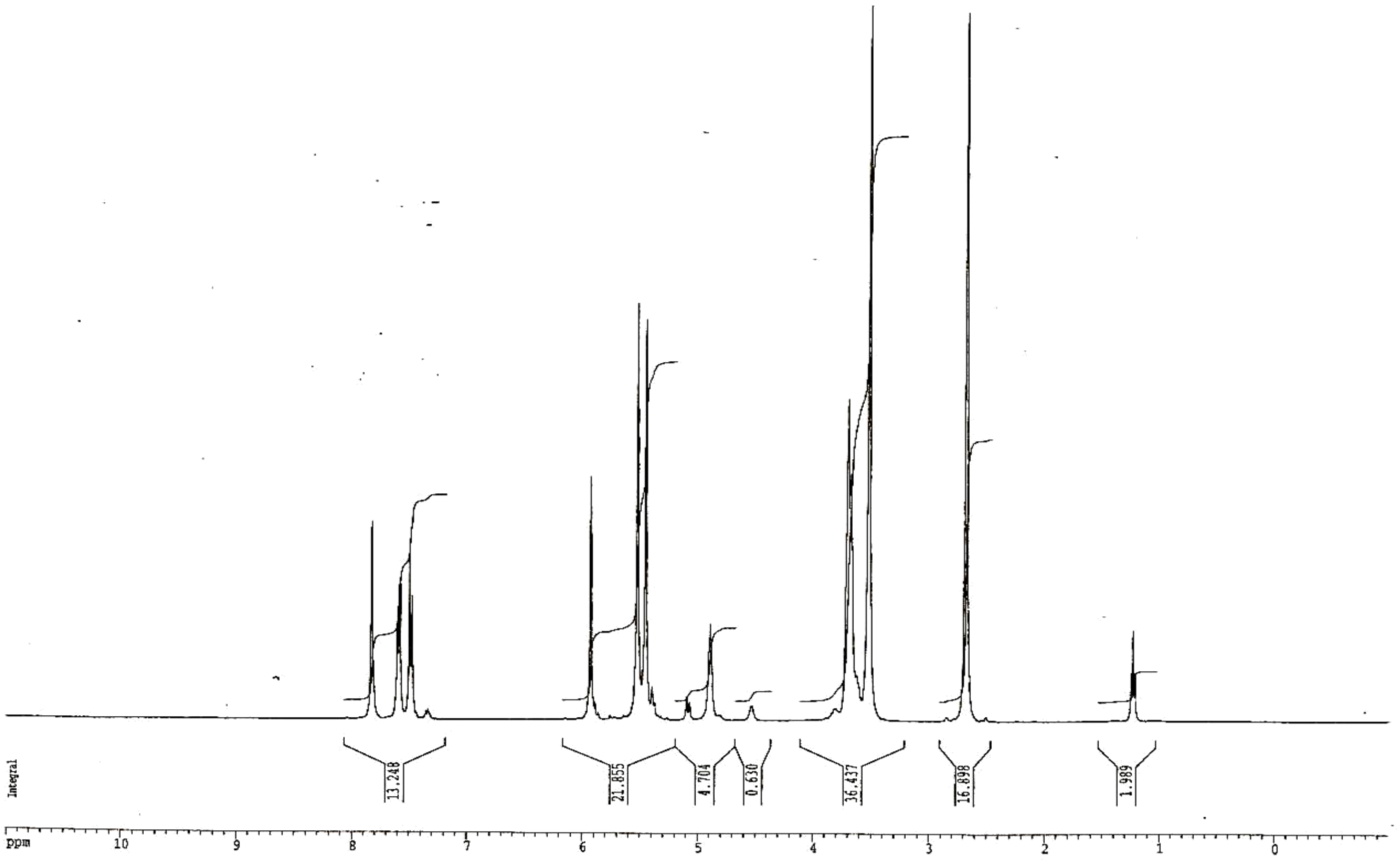

Current AMX400 Data
DU /x
NAME 20070606
EXPNO 90
PROCNO 1

F2 - Acquisition Parameters
Date_ 20070606
Time 17.46
INSTRUM spect
PROBHD 5 mm Dual 13
PULPROG zg30
TD 65536
SOLVENT DMSO
NS 16
DS 2
SWH 8333.373 Hz
FIDRES 0.127157 Hz
AQ 3.9322100 sec
RG 512
DW 60.000 usec
DE 85.71 usec
TE 300.0 K
HL1 1 dB
D1 1.00000000 sec
P1 12.50 usec
SFO1 400.1387237 MHz
NUCLEUS 1H

F2 - Processing parameters
SI 32768
SF 400.1362172 MHz
WDW EM
SSB 0
LB 0.30 Hz
GB 0
PC 1.00

1D NMR plot parameters
CX 30.00 cm
F1P 11.000 ppm
F1 4401.50 Hz
F2P -1.000 ppm
F2 -400.14 Hz
PPMCM 0.40000 ppm/cm
HZCM 160.05449 Hz/cm

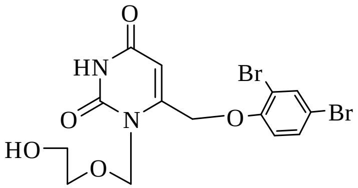

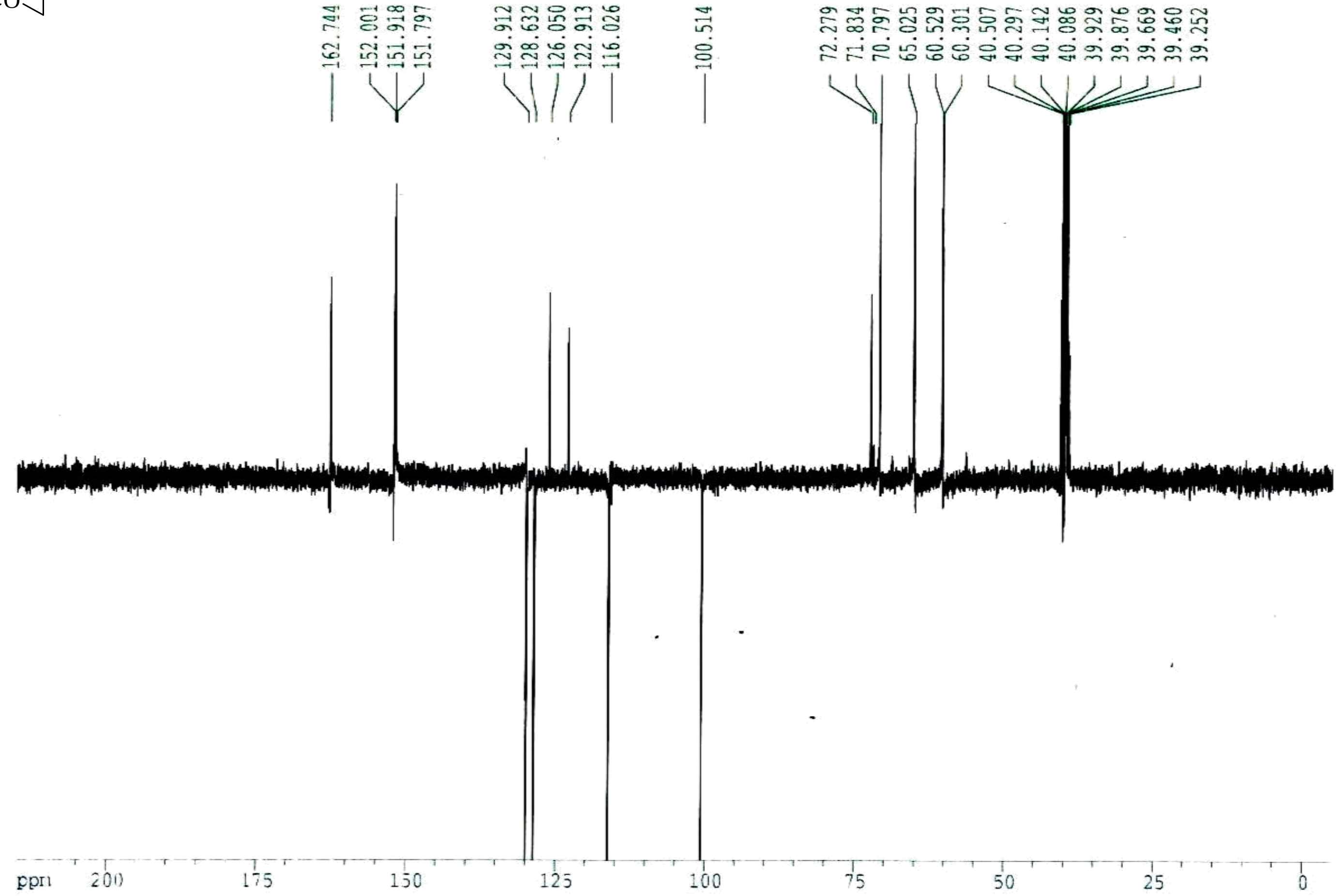

Current AMX400 Data

DU	/x	
NAME	20070608	
EXPNO	200	
PROCNO	1	

F2 - Acquisition Parameters

Date_	20070608	
Time	23.02	
INSTRUM	spect	
PROBHD	5 mm Dual 13	
PULPROG	pendant.amx	
TD	65536	
SOLVENT	DMSO	
NS	800	
DS	4	
SWH	25000.119	Hz
FIDRES	0.381472	Hz
AQ	1.3107700	sec
RG	32768	
DW	20.000	usec
DE	28.57	usec
TE	300.0	K
P1	6.50	usec
P3	10.60	usec
CNST2	145.0000000	
HL1	1	dB
D1	1.50000000	sec
S1	1	dB
SFO2	400.1379006	MHz
DECNUC	1H	
D4	0.0017241	sec
P4	21.2	usec
P2	13.0	usec
D15	0.0043103	sec
D13	0.0000040	sec
S2	26	dB
SFO1	100.6245885	MHz
NUCLEUS	13C	
CPDPRG	waltz16	
P31	90.00	usec
D2	0.00357143	sec

F2 - Processing parameters

SI	32768	
SF	100.6143589	MHz
WDW	EM	
SSB	0	
LB	1.00	Hz
GB	0	
PC	1.40	

1D NMR plot parameters

CX	20.00	cm
F1P	215.000	ppm
F1	21632.09	Hz
F2P	-5.000	ppm
F2	-503.07	Hz
PPMCM	11.00000	ppm/cm
HZCM	1106.75793	Hz/cm

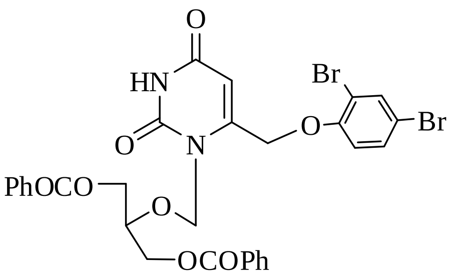

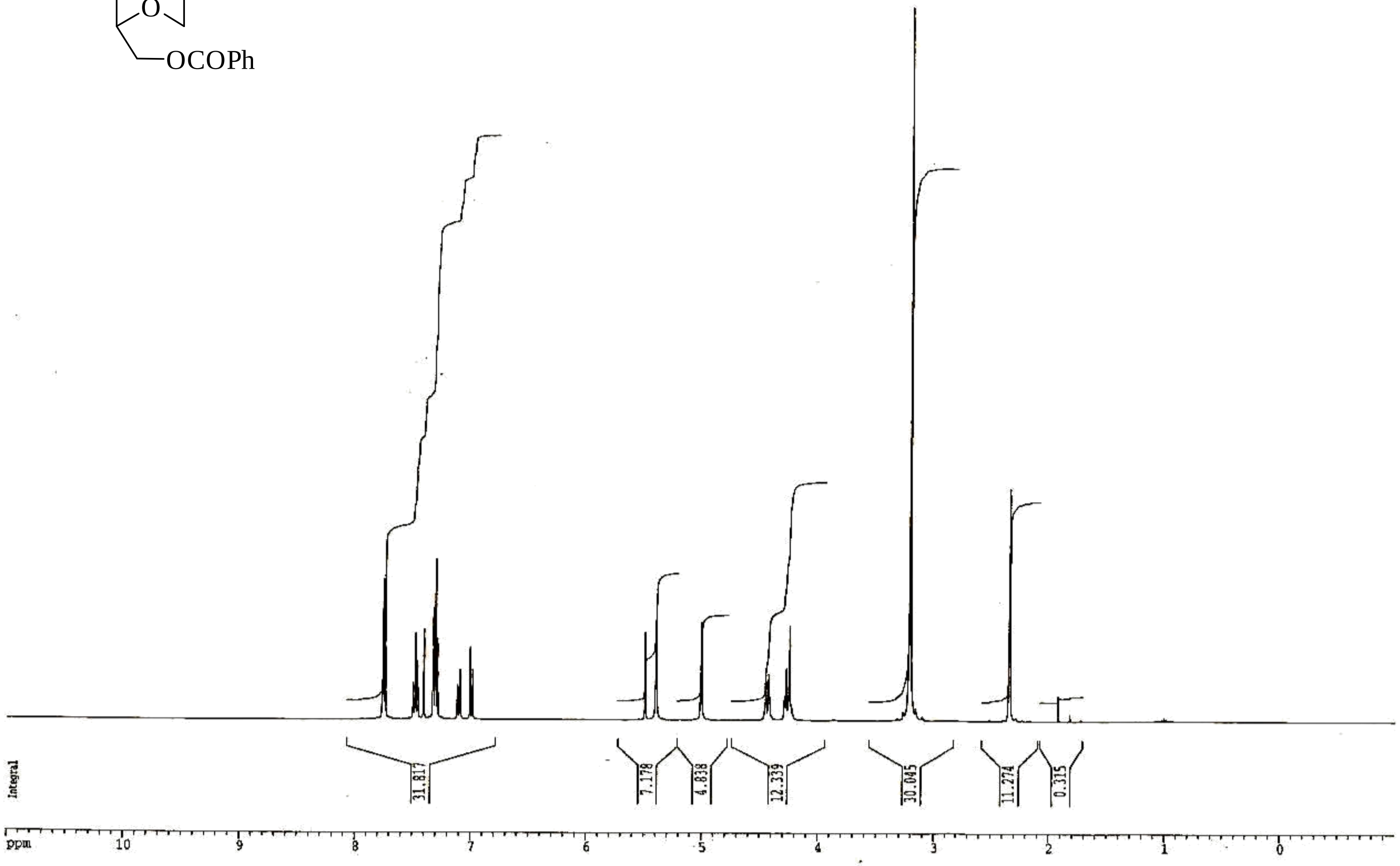

Current AMX400 Data
DU /x
NAME 20070821
EXPNO 200
PROCNO 1

F2 - Acquisition Parameters
Date_ 20070822
Time 6.48
INSTRUM spect
PROBHD 5 mm Dual 13
PULPROG zg30
TD 65536
SOLVENT DMSO
NS 16
DS 2
SWH 8333.373 Hz
FIDRES 0.127157 Hz
AQ 3.9322100 sec
RG 256
DW 60.000 usec
DE 85.71 usec
TE 300.0 K
HL1 1 dB
D1 1.00000000 sec
P1 12.50 usec
SFO1 400.1387237 MHz
NUCLEUS 1H

F2 - Processing parameters
SI 32768
SF 400.1363524 MHz
WDW EM
SSB 0
LB 0.30 Hz
GB 0
PC 1.00

1D NMR plot parameters
CX 30.00 cm
F1P 11.000 ppm
F1 4401.50 Hz
F2P -1.000 ppm
F2 -400.14 Hz
PPMCM 0.40000 ppm/cm
HZCM 160.05453 Hz/cm

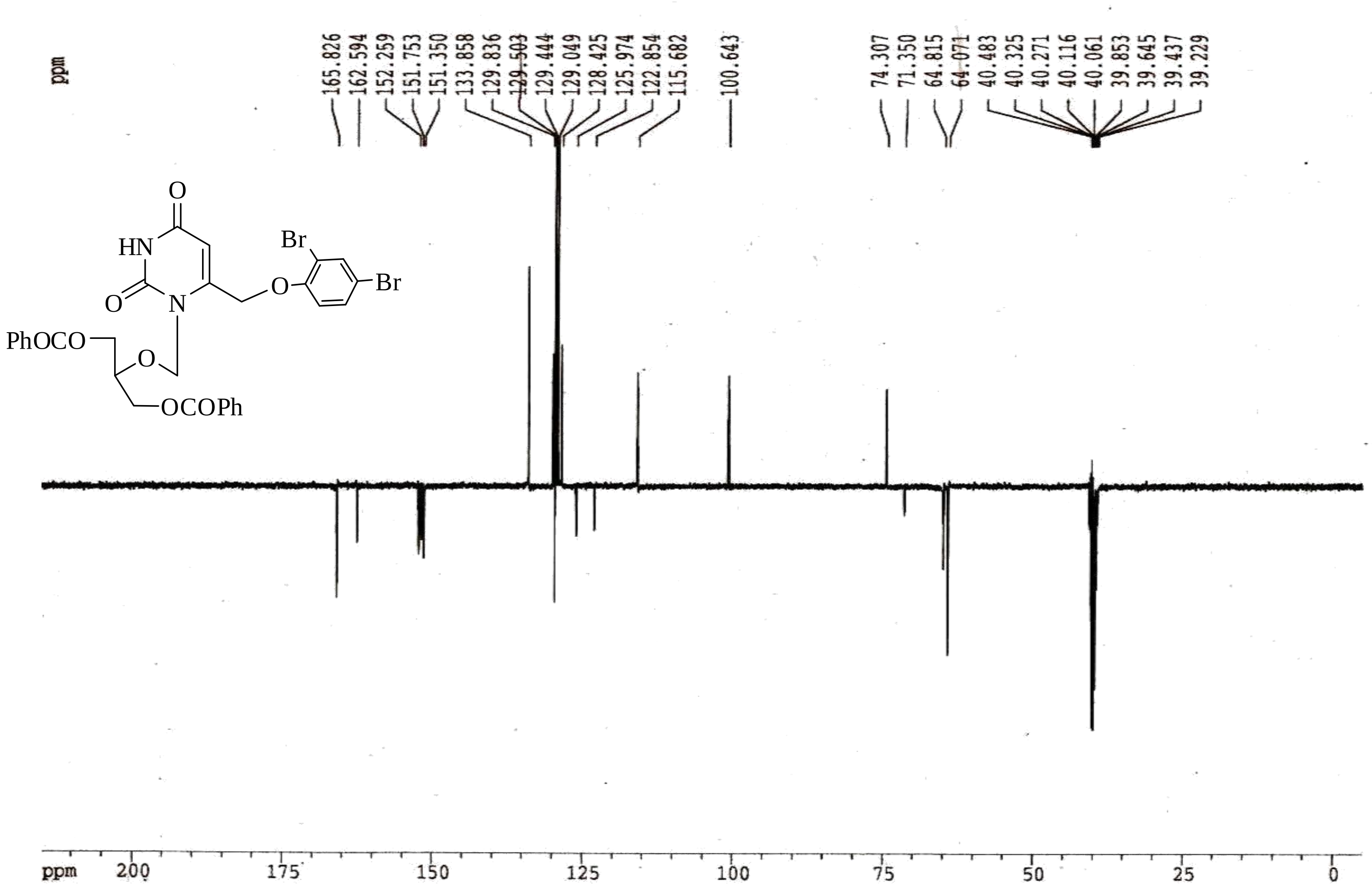

Current AMX400 Data

DU	/x
NAME	20070823
EXPNO	200
PROCNO	1

F2 - Acquisition Parameters

Date_	20070823
Time	19.10
INSTRUM	spect
PROBHD	5 mm Dual 13
PULPROG	pendant.amx
TD	65536
SOLVENT	DMSO
NS	800
DS	4
SWH	25000.119 Hz
FIDRES	0.381472 Hz
AQ	1.3107700 sec
RG	32768
DW	20.000 usec
DE	28.57 usec
TE	300.0 K
P1	6.50 usec
P3	10.60 usec
CNST2	145.0000000
HL1	1 dB
D1	1.50000000 sec
S1	1 dB
SFO2	400.1379006 MHz
DECNUC	1H
D4	0.0017241 sec
P4	21.2 usec
P2	13.0 usec
D15	0.0043103 sec
D13	0.0000040 sec
S2	26 dB
SFO1	100.6245885 MHz
NUCLEUS	13C
CPDPRG	waltz16
P31	90.00 usec
D2	0.00357143 sec

F2 - Processing parameters

SI	32768
SF	100.6143589 MHz
WDW	EM
SSB	0
LB	1.00 Hz
GB	0
PC	1.40

1D NMR plot parameters

CX	20.00 cm
F1P	215.000 ppm
F1	21632.09 Hz
F2P	-5.000 ppm
F2	-503.07 Hz
PPMCM	11.00000 ppm/cm
HZCM	1106.75793 Hz/cm

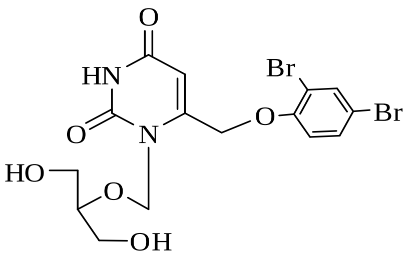

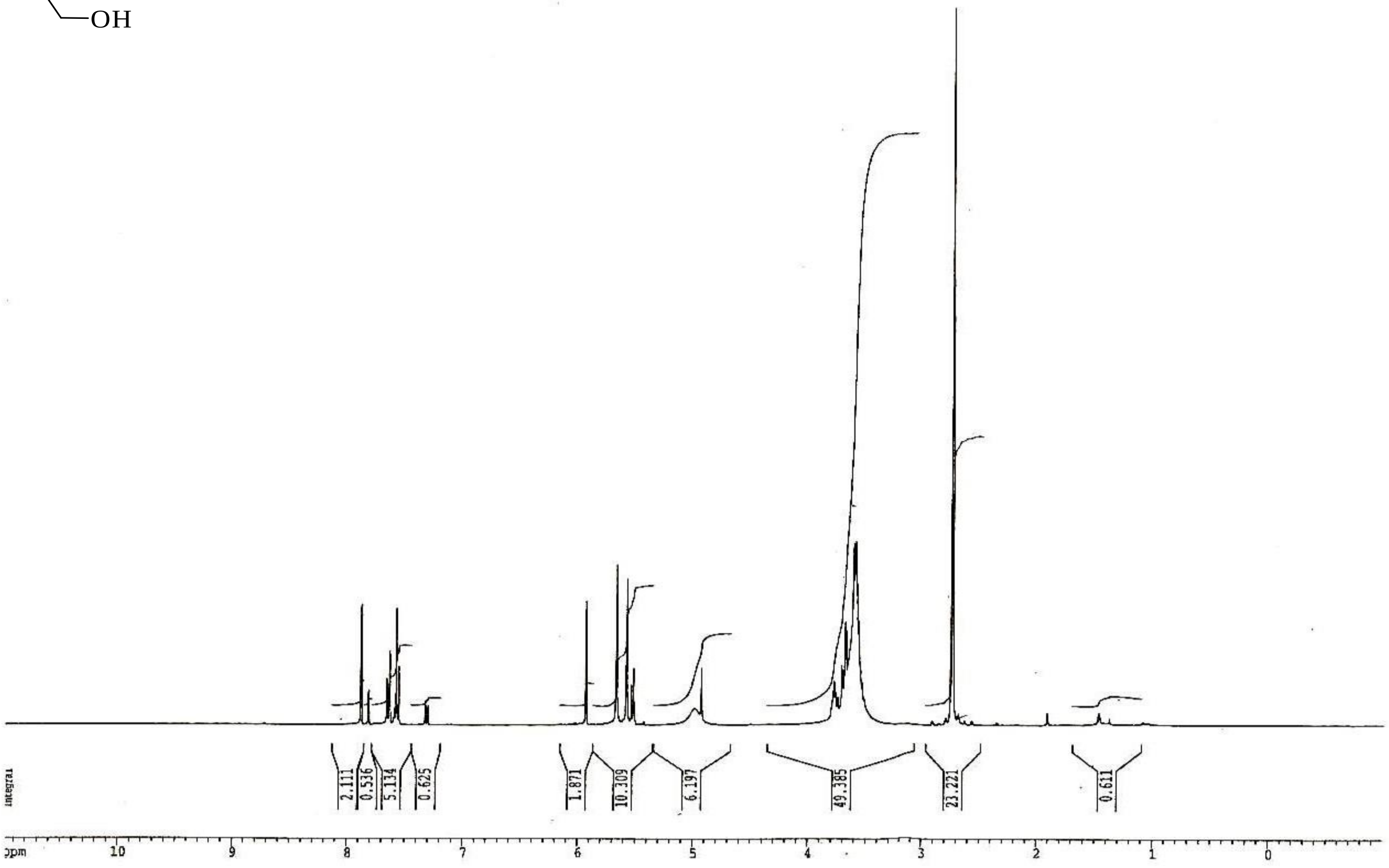


```
Current AMX400 Data
DU                      /x
NAME              20071002
EXPNO                  170
PROCNO                   1

F2 - Acquisition Parameters
Date_             20071003
Time                  1.09
INSTRUM              spect
PROBHD       5 mm Dual 13
PULPROG               zg30
TD                   65536
SOLVENT               DMSO
NS                      16
DS                       2
SWH              8333.373 Hz
FIDRES           0.127157 Hz
AQ              3.9322100 sec
RG                     512
DW                  60.000 usec
DE                   85.71 usec
TE                   300.0 K
HL1                      1 dB
D1              1.00000000 sec
P1                   12.50 usec
SFO1           400.1387237 MHz
NUCLEUS                 1H

F2 - Processing parameters
SI                   32768
SF             400.1361953 MHz
WDW                     EM
SSB                      0
LB                    0.30 Hz
GB                       0
PC                    1.00

1D NMR plot parameters
CX                   30.00 cm
F1P                 11.000 ppm
F1                 4401.50 Hz
F2P                 -1.000 ppm
F2                 -400.14 Hz
PPMCM              0.40000 ppm/cm
HZCM             160.05447 Hz/cm
```

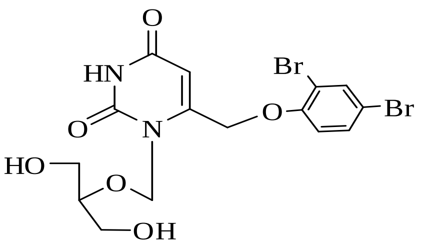

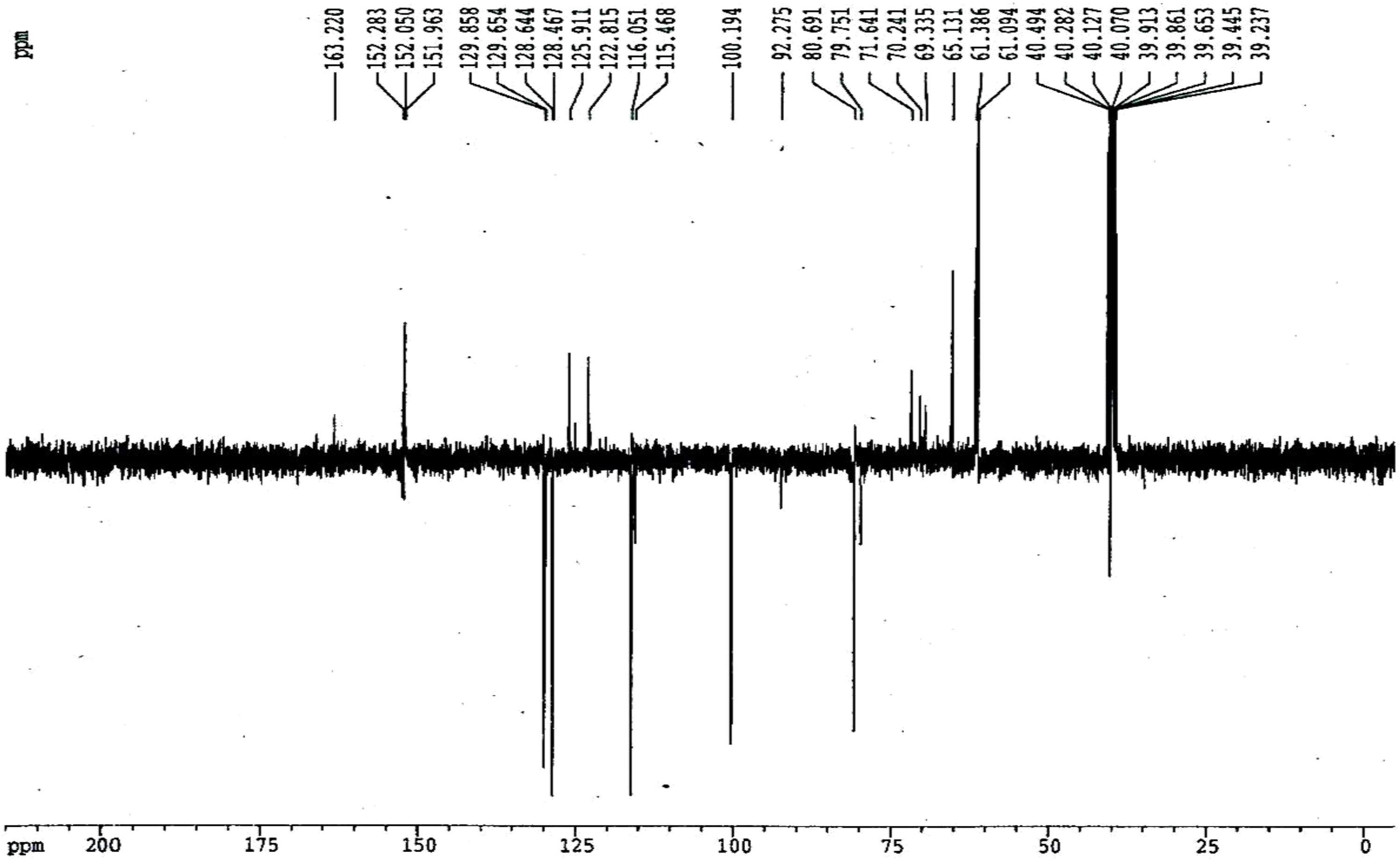

Current AMX400 Data

DU	/x	
NAME	20071004	
EXPNO	80	
PROCNO	1	

F2 - Acquisition Parameters

Date_	20071004	
Time	14.29	
INSTRUM	spect	
PROBHD	5 mm Dual 13	
PULPROG	pendant.amx	
TD	65536	
SOLVENT	DMSO	
NS	800	
DS	4	
SWH	25000.119	Hz
FIDRES	0.381472	Hz
AQ	1.3107700	sec
RG	32768	
DW	20.000	usec
DE	28.57	usec
TE	300.0	K
P1	6.50	usec
P3	10.60	usec
CNST2	145.0000000	
HL1	1	dB
D1	1.50000000	sec
S1	1	dB
SFO2	400.1379006	MHz
DECNUC	1H	
D4	0.0017241	sec
P4	21.2	usec
P2.	13.0	usec
D15	0.0043103	sec
D13	0.0000040	sec
S2	26	dB
SFO1	100.6245885	MHz
NUCLEUS	13C	
CPDPRG	waltz16	
P31	90.00	usec
D2	0.00357143	sec

F2 - Processing parameters

SI	32768	
SF	100.6143589	MHz
WDW	EM	
SSB	0	
LB	1.00	Hz
GB	0	
PC	1.40	

1D NMR plot parameters

CX	20.00	cm
F1P	215.000	ppm
F1	21632.09	Hz
F2P	-5.000	ppm
F2	-503.07	Hz
PPMCM	11.00000	ppm/c[illegible]
HZCM	1106.75793	Hz/cm

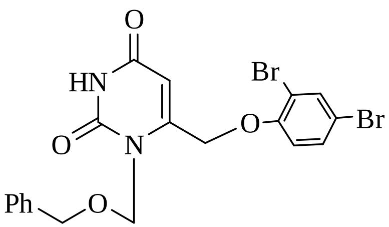

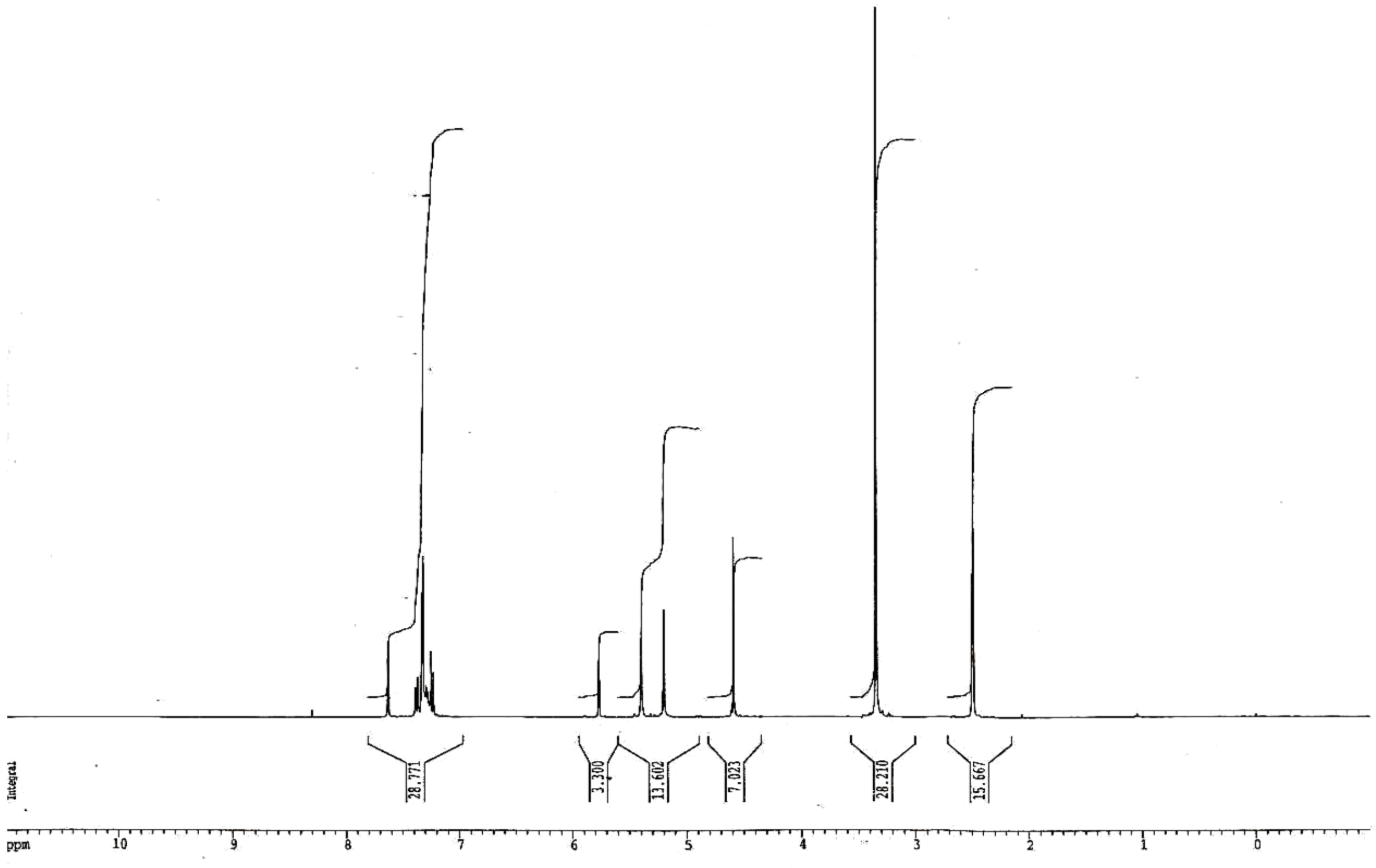

Current AMX400 Data

DU	/x
NAME	20070524
EXPNO	210
PROCNO	1

F2 - Acquisition Parameters

Date_	20070524
Time	22.00
INSTRUM	spect
PROBHD	5 mm Dual 13
PULPROG	zg30
TD	65536
SOLVENT	DMSO
NS	16
DS	2
SWH	8333.373 Hz
FIDRES	0.127157 Hz
AQ	3.9322100 sec
RG	512
DW	60.000 usec
DE	85.71 usec
TE	300.0 K
HL1	1 dB
D1	1.00000000 sec
P1	12.50 usec
SFO1	400.1387237 MHz
NUCLEUS	1H

F2 - Processing parameters

SI	32768
SF	400.1362922 MHz
WDW	EM
SSB	0
LB	0.30 Hz
GB	0
PC	1.00

1D NMR plot parameters

CX	30.00 cm
F1P	11.000 ppm
F1	4401.50 Hz
F2P	-1.000 ppm
F2	-400.14 Hz
PPMCM	0.40000 ppm/cm
HZCM	160.05452 Hz/cm

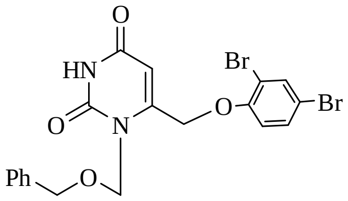

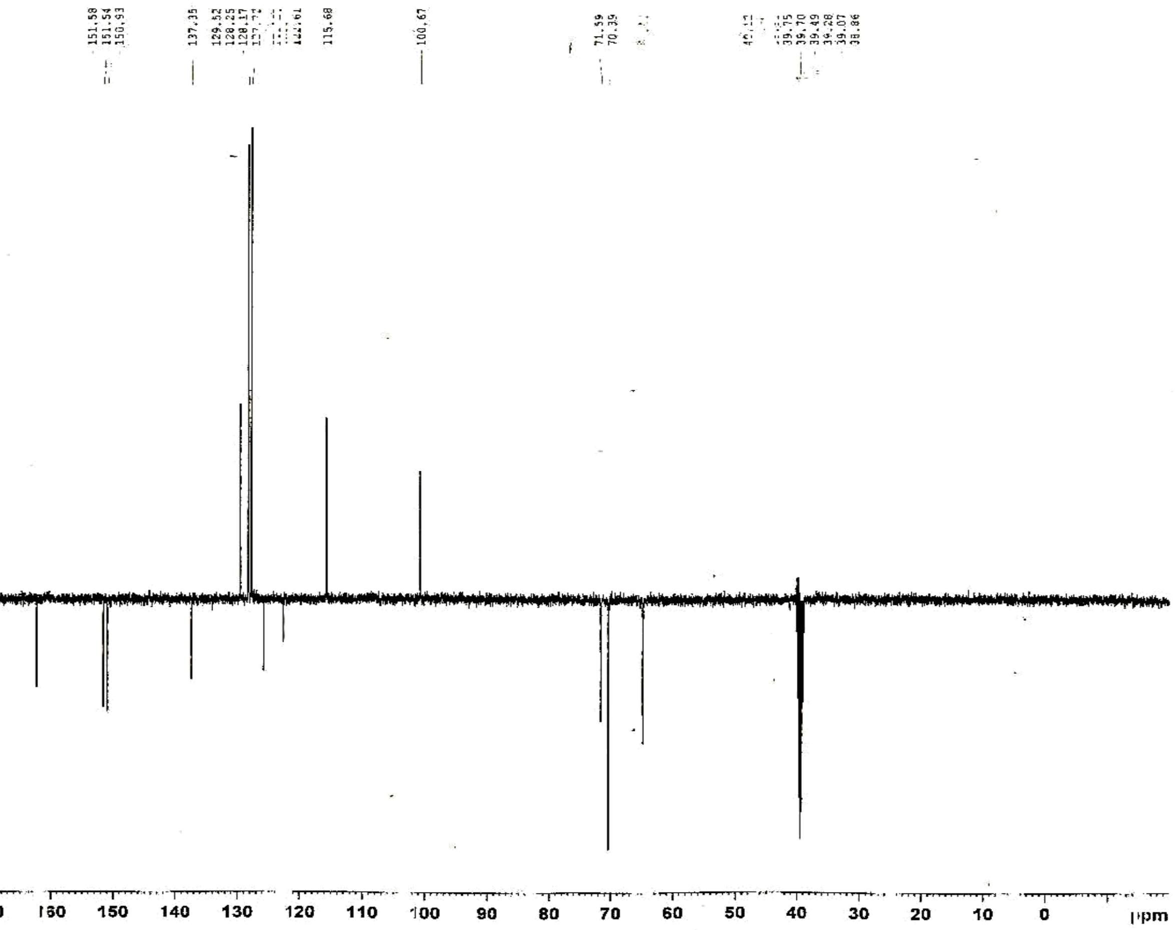

BRUKER

Current AV400 Data
NAME 31052007-nmr
EXPNO 680
PROCNO 1

F2 - Acquisition Parameters
Date_ 20070531
Time 11.47
INSTRUM spect
PROBHD 5 mm PABBO BB-
PULPROG jmod
TD 65536
SOLVENT DMSO
NS 464
DS 4
SWH 24038.461 Hz
FIDRES 0.366798 Hz
AQ 1.3631988 sec
RG 2050
DW 20.800 usec
DE 6.00 usec
TE 298.7 K
CNST2 145.0000000
CNST11 1.0000000
D1 1.00000000 sec
d20 0.00689655 sec
DELTA 0.00001222 sec
TD0 1

======== CHANNEL f1 ========
NUC1 13C
P1 9.60 usec
p2 19.20 usec
PL1 -1.00 dB
SFO1 100.6530068 MHz

======== CHANNEL f2 ========
CPDPRG2 waltz16
NUC2 1H
PCPD2 80.00 usec
PL2 0.00 dB
PL12 14.20 dB
SFO2 400.2516010 MHz

F2 - Processing parameters
SI 32768
SF 100.6429933 MHz
WDW EM
SSB 0
LB 1.00 Hz
GB 0
PC 1.40

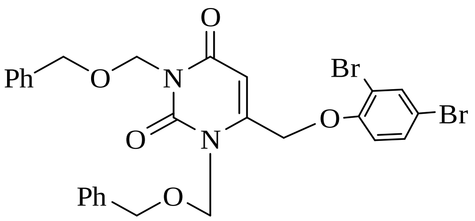

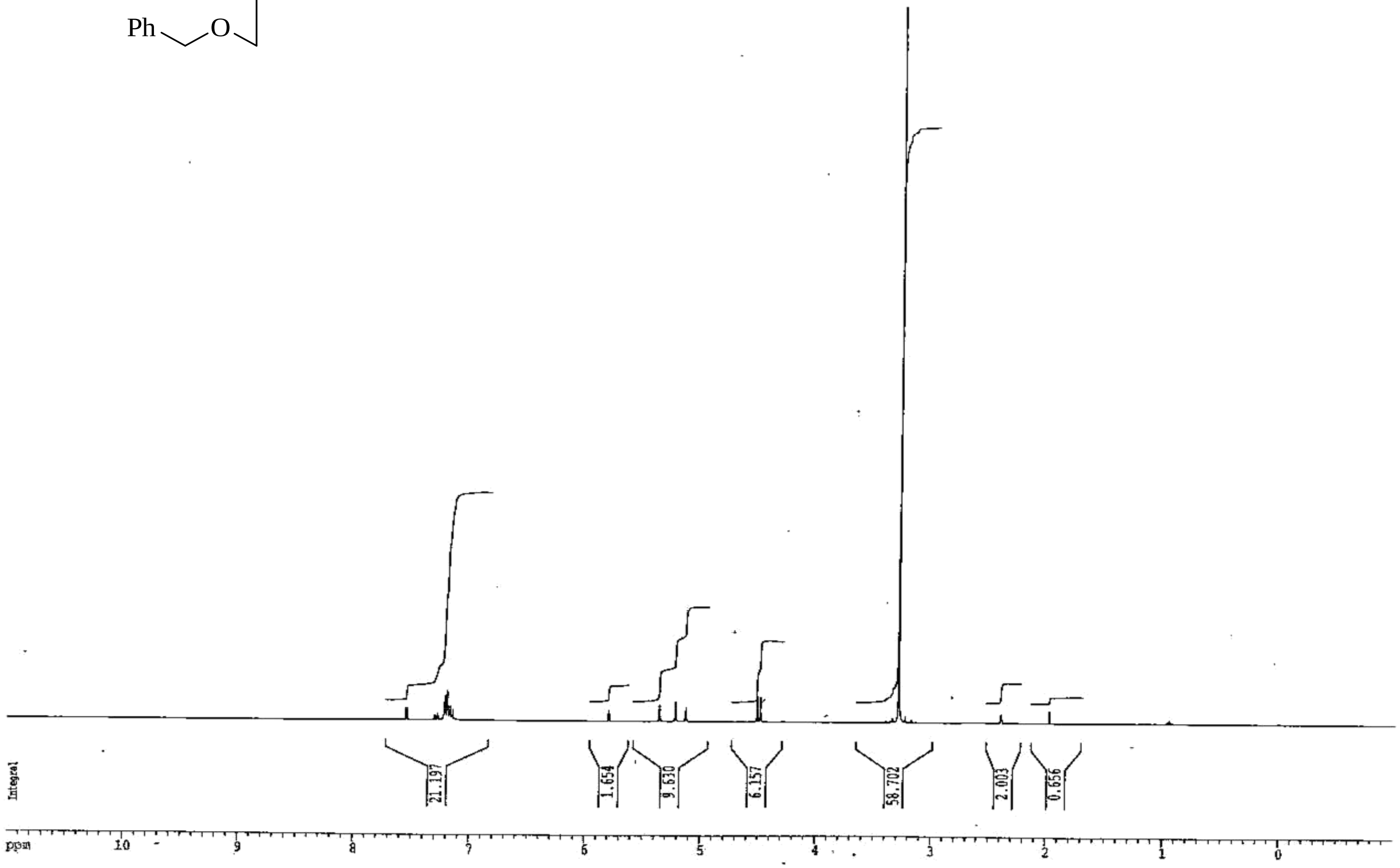

Current AMX400 Data
DU /x
NAME 20070425
EXPNO 130
PROCNO 1

F2 - Acquisition Parameters
Date_ 20070425
Time 21.19
INSTRUM spect
PROBHD 5 mm Dual 13
PULPROG zg30
TD 65536
SOLVENT DMSO
NS 16
DS 2
SWH 8333.373 Hz
FIDRES 0.127157 Hz
AQ 3.9322100 sec
RG 256
DW 60.000 usec
DE 85.71 usec
TE 300.0 K
HL1 1 dB
D1 1.00000000 sec
P1 12.50 usec
SFO1 400.1387237 MHz
NUCLEUS 1H

F2 - Processing parameters
SI 32768
SF 400.1363318 MHz
WDW EM
SSB 0
LB 0.30 Hz
GB 0
PC 1.00

1D NMR plot parameters
CX 30.00 cm
F1P 11.000 ppm
F1 4401.50 Hz
F2P -1.000 ppm
F2 -400.14 Hz
PPMCM 0.40000 ppm/cm
HZCM 160.05453 Hz/cm

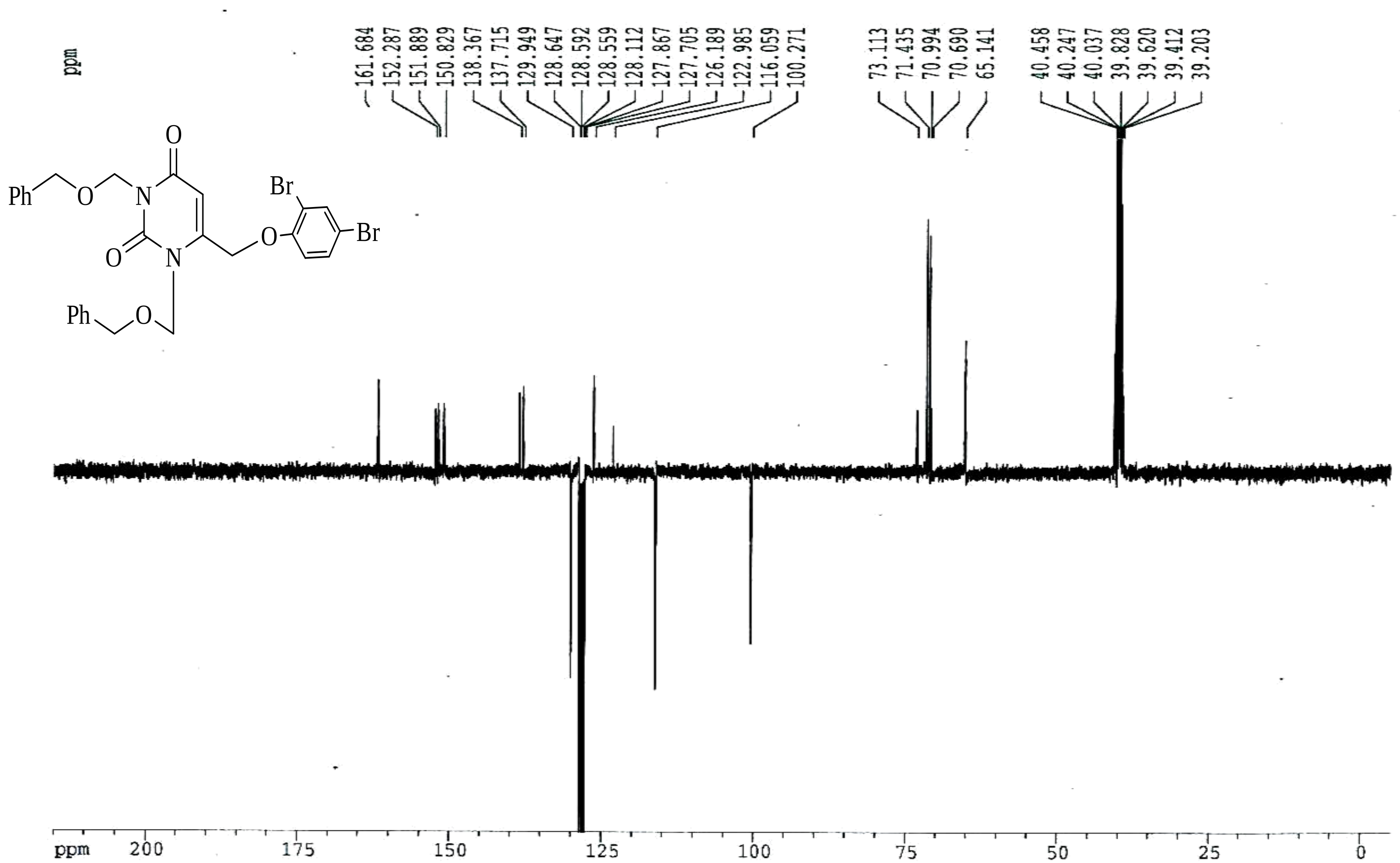


```
Current AMX400 Data
DU                          /x
NAME                  20070424
EXPNO                      430
PROCNO                       1

F2 - Acquisition Paramete
Date_                 20070425
Time                      8.38
INSTRUM                  spect
PROBHD           5 mm Dual 13
PULPROG           pendant.amx
TD                       65536
SOLVENT                   DMSO
NS                         800
DS                           4
SWH                  25000.119 Hz
FIDRES                0.381472 Hz
AQ                   1.3107700 sec
RG                       32768
DW                      20.000 usec
DE                       28.57 usec
TE                       300.0 K
P1                        6.50 usec
P3                       10.60 usec
CNST2              145.0000000
HL1                          1 dB
D1                  1.50000000 sec
S1                           1 dB
SFO2               400.1379006 MHz
DECNUC                      1H
D4                   0.0017241 sec
P4                        21.2 usec
P2                        13.0 usec
D15                  0.0043103 sec
D13                  0.0000040 sec
S2                          26 dB
SFO1               100.6245885 MHz
NUCLEUS                    13C
CPDPRG                 waltz16
P31                      90.00 usec
D2                  0.00357143 sec

F2 - Processing parameters
SI                       32768
SF                 100.6143589 MHz
WDW                         EM
SSB                          0
LB                        1.00 Hz
GB                           0
PC                        1.40

1D NMR plot parameters
CX                       20.00 cm
F1P                    215.000 ppm
F1                    21632.09 Hz
F2P                     -5.000 ppm
F2                     -503.07 Hz
PPMCM                 11.00000 ppm/cm
HZCM                1106.75793 Hz/cm
```

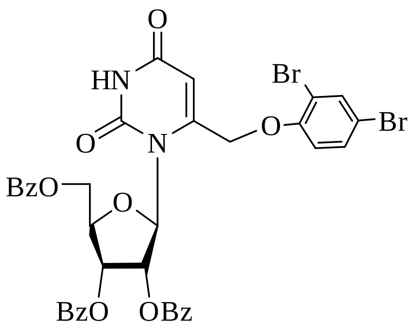

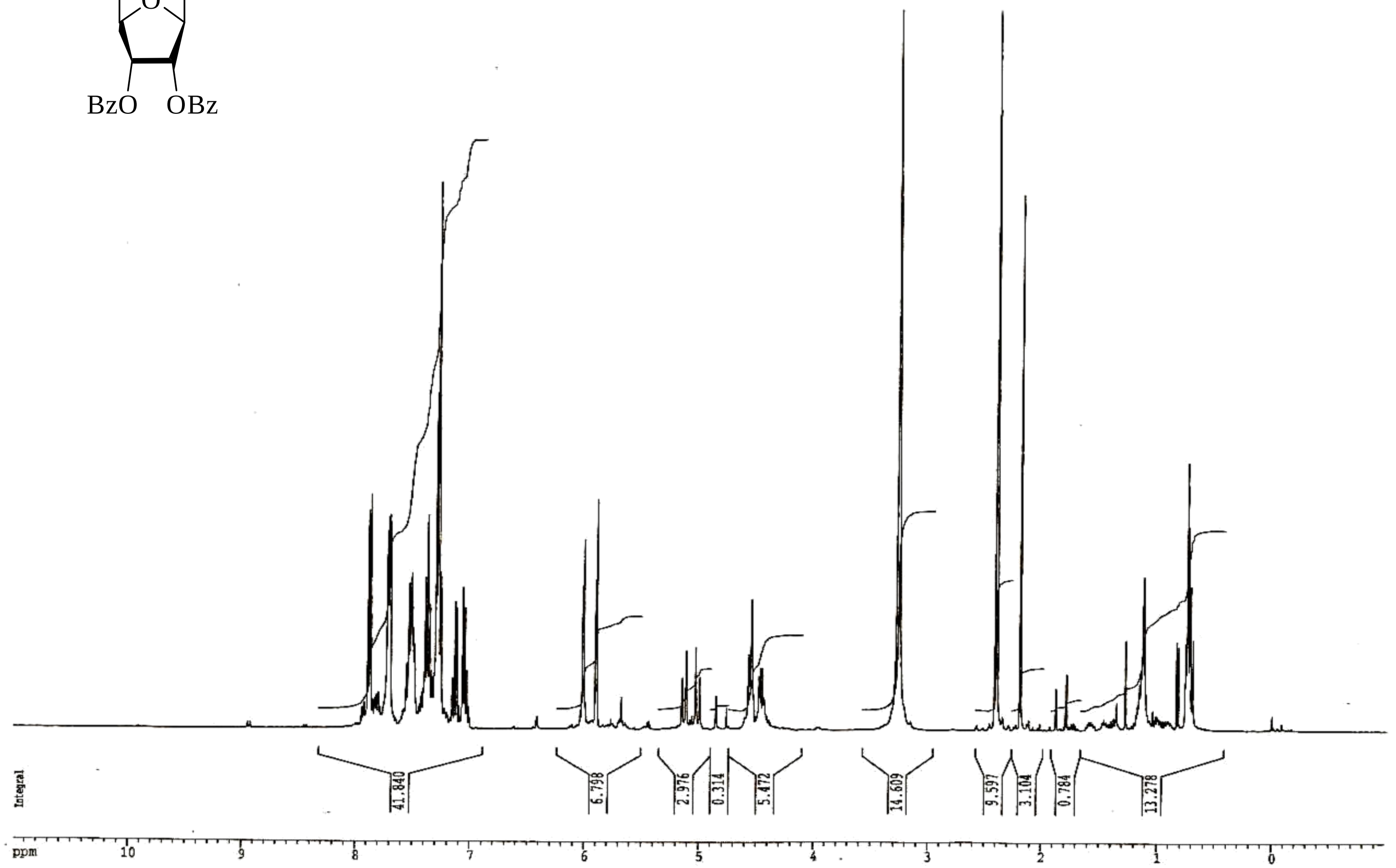

Current AMX400 Data

DU	/x
NAME	20070511
EXPNO	410
PROCNO	1

F2 - Acquisition Parameters

Date_	20070511
Time	22.21
INSTRUM	spect
PROBHD	5 mm Dual 13
PULPROG	zg30
TD	65536
SOLVENT	DMSO
NS	16
DS	2
SWH	8333.373 Hz
FIDRES	0.127157 Hz
AQ	3.9322100 sec
RG	256
DW	60.000 usec
DE	85.71 usec
TE	300.0 K
HL1	1 dB
D1	1.00000000 sec
P1	12.50 usec
SFO1	400.1387237 MHz
NUCLEUS	1H

F2 - Processing parameters

SI	32768
SF	400.1363282 MHz
WDW	EM
SSB	0
LB	0.30 Hz
GB	0
PC	1.00

1D NMR plot parameters

CX	30.00 cm
F1P	11.000 ppm
F1	4401.50 Hz
F2P	-1.000 ppm
F2	-400.14 Hz
PPMCM	0.40000 ppm/cm
HZCM	160.05453 Hz/cm

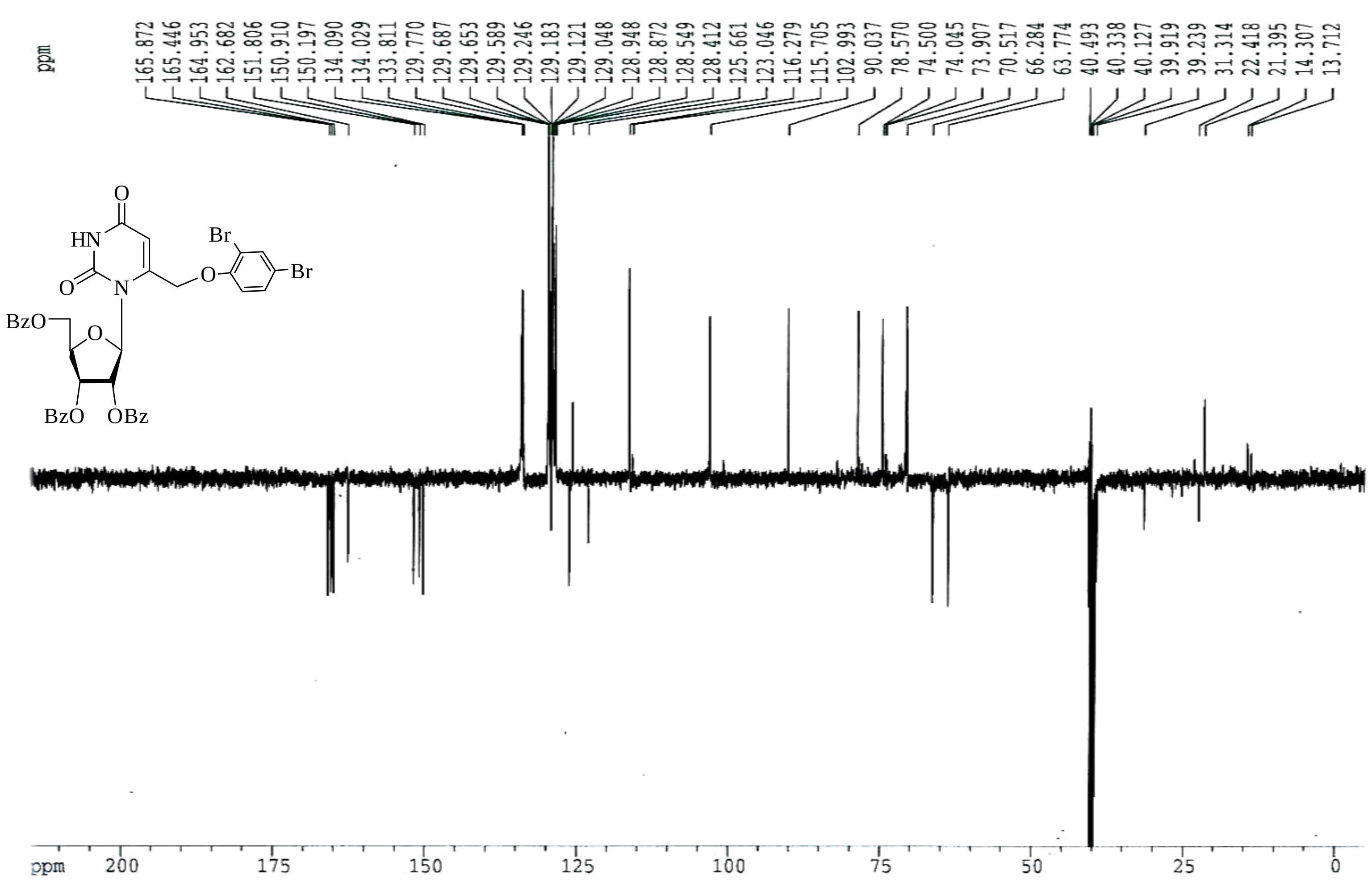

Current AMX400 Data
DU /x
NAME 20070514
EXPNO 180
PROCNO 1

F2 - Acquisition Parameters
Date_ 20070514
Time 18.08
INSTRUM spect
PROBHD 5 mm Dual 13
PULPROG pendant.amx
TD 65536
SOLVENT DMSO
NS 800
DS 4
SWH 25000.119 Hz
FIDRES 0.381472 Hz
AQ 1.3107700 sec
RG 32768
DW 20.000 usec
DE 28.57 usec
TE 300.0 K
P1 6.50 usec
P3 10.60 usec
CNST2 145.0000000
HL1 1 dB
D1 1.50000000 sec
S1 1 dB
SFO2 400.1379006 MHz
DECNUC 1H
D4 0.0017241 sec
P4 21.2 usec
P2 13.0 usec
D15 0.0043103 sec
D13 0.0000040 sec
S2 26 dB
SFO1 100.6245885 MHz
NUCLEUS 13C
CPDPRG waltz16
P31 90.00 usec
D2 0.00357143 sec

F2 - Processing parameters
SI 32768
SF 100.6143589 MHz
WDW EM
SSB 0
LB 1.00 Hz
GB 0
PC 1.40

1D NMR plot parameters
CX 20.00 cm
F1P 215.000 ppm
F1 21632.09 Hz
F2P -5.000 ppm
F2 -503.07 Hz
PPMCM 11.00000 ppm/cm
HZCM 1106.75793 Hz/cm

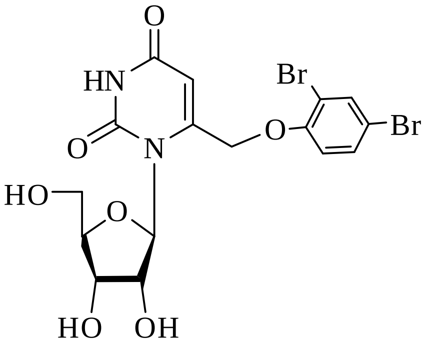

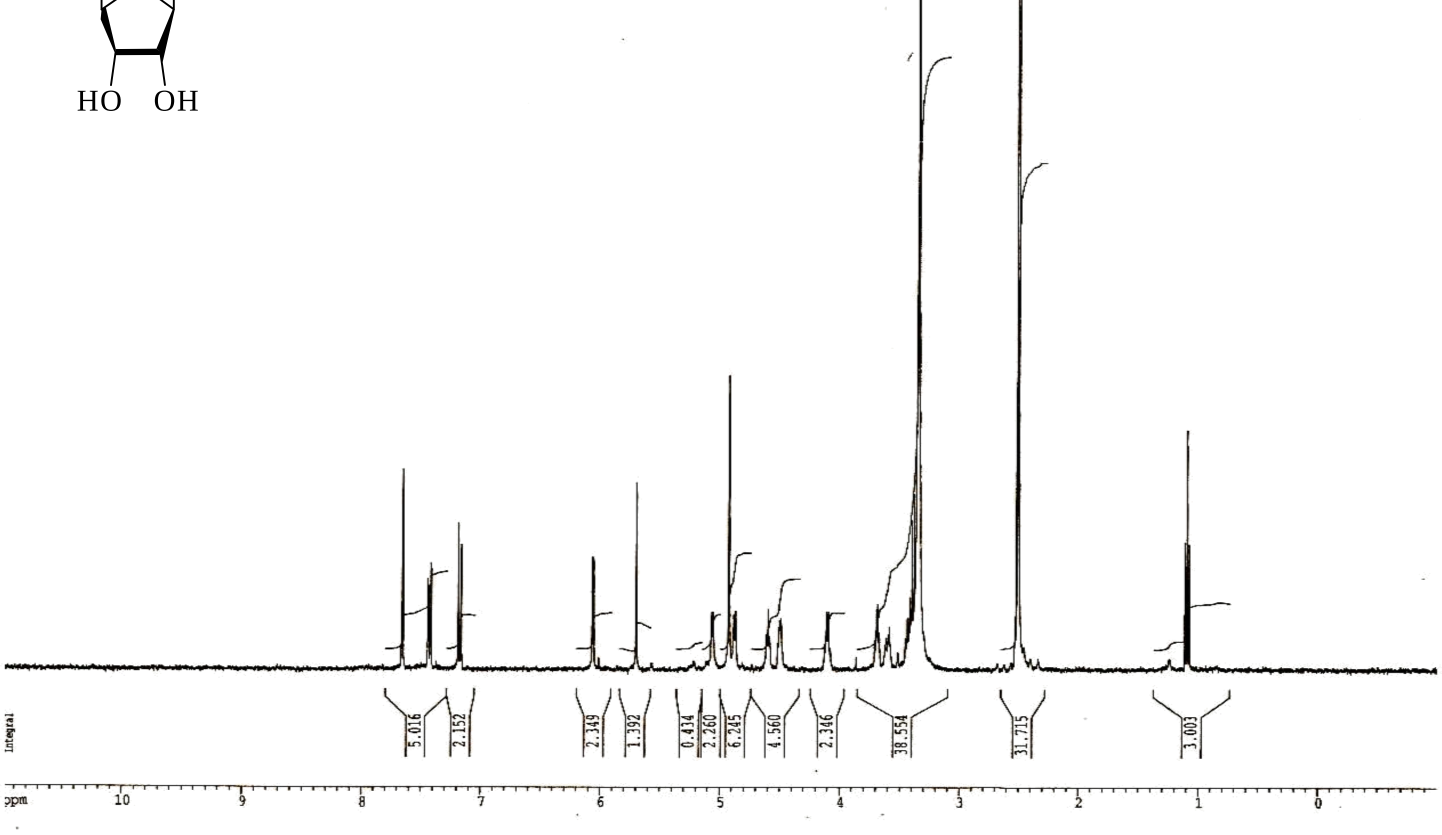

Current AMX400 Data
DU /x
NAME 20071017
EXPNO 100
PROCNO 1

F2 - Acquisition Parameters
Date_ 20071017
Time 23.00
INSTRUM spect
PROBHD 5 mm Dual 13
PULPROG zg30
TD 65536
SOLVENT DMSO
NS 16
DS 2
SWH 8333.373 Hz
FIDRES 0.127157 Hz
AQ 3.9322100 sec
RG 512
DW 60.000 usec
DE 85.71 usec
TE 300.0 K
HL1 1 dB
D1 1.00000000 sec
P1 12.50 usec
SFO1 400.1387237 MHz
NUCLEUS 1H

F2 - Processing parameters
SI 32768
SF 400.1362838 MHz
WDW EM
SSB 0
LB 0.30 Hz
GB 0
PC 1.00

1D NMR plot parameters
CX 30.00 cm
F1P 11.000 ppm
F1 4401.50 Hz
F2P -1.000 ppm
F2 -400.14 Hz
PPMCM 0.40000 ppm/cm
HZCM 160.05452 Hz/cm

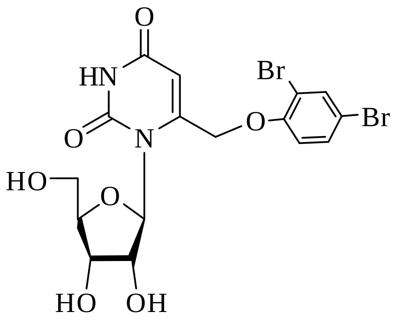

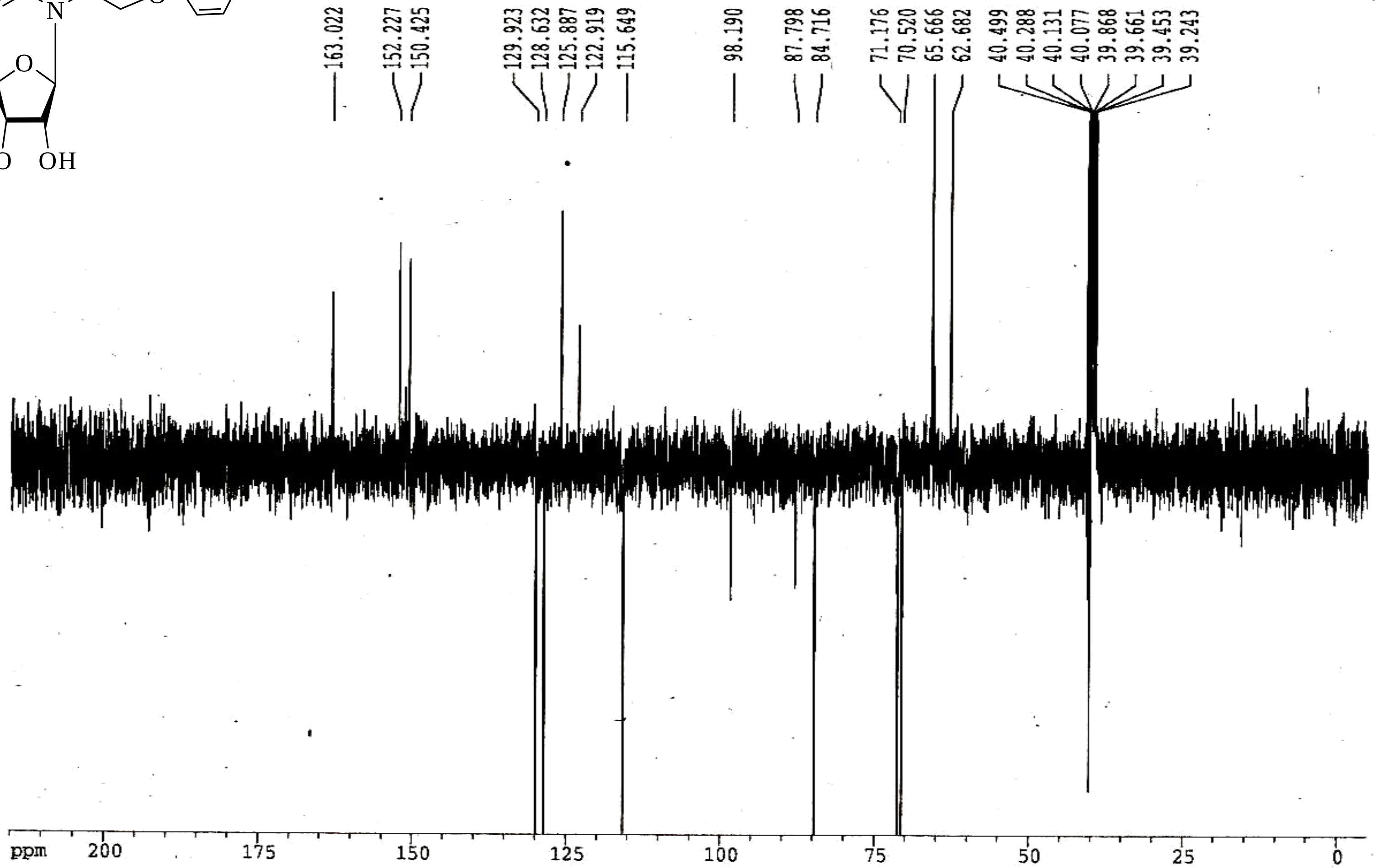

Current AMX400 Data
DU /x
NAME 20071019
EXPNO 230
PROCNO 1

F2 - Acquisition Parameters
Date_ 20071019
Time 18.36
INSTRUM spect
PROBHD 5 mm Dual 13
PULPROG pendant.amx
TD 65536
SOLVENT DMSO
NS 800
DS 4
SWH 25000.119 Hz
FIDRES 0.381472 Hz
AQ 1.3107700 sec
RG 32768
DW 20.000 usec
DE 28.57 usec
TE 300.0 K
P1 6.50 usec
P3 10.60 usec
CNST2 145.0000000
HL1 1 dB
D1 1.50000000 sec
S1 1 dB
SFO2 400.1379006 MHz
DECNUC 1H
D4 0.0017241 sec
P4 21.2 usec
P2 13.0 usec
D15 0.0043103 sec
D13 0.0000040 sec
S2 26 dB
SFO1 100.6245885 MHz
NUCLEUS 13C
CPDPRG waltz16
P31 90.00 usec
D2 0.00357143 sec

F2 - Processing parameters
SI 32768
SF 100.6143589 MHz
WDW EM
SSB 0
LB 1.00 Hz
GB 0
PC 1.40

1D NMR plot parameters
CX 20.00 cm
F1P 215.000 ppm
F1 21632.09 Hz
F2P -5.000 ppm
F2 -503.07 Hz
PPMCM 11.00000 ppm/cm
HZCM 1106.75793 Hz/cm

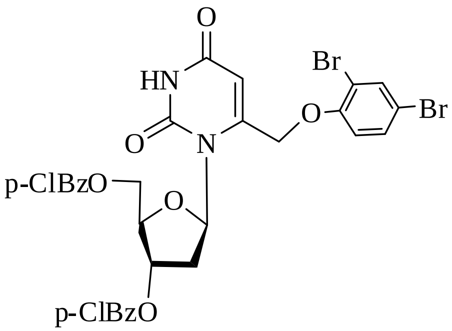

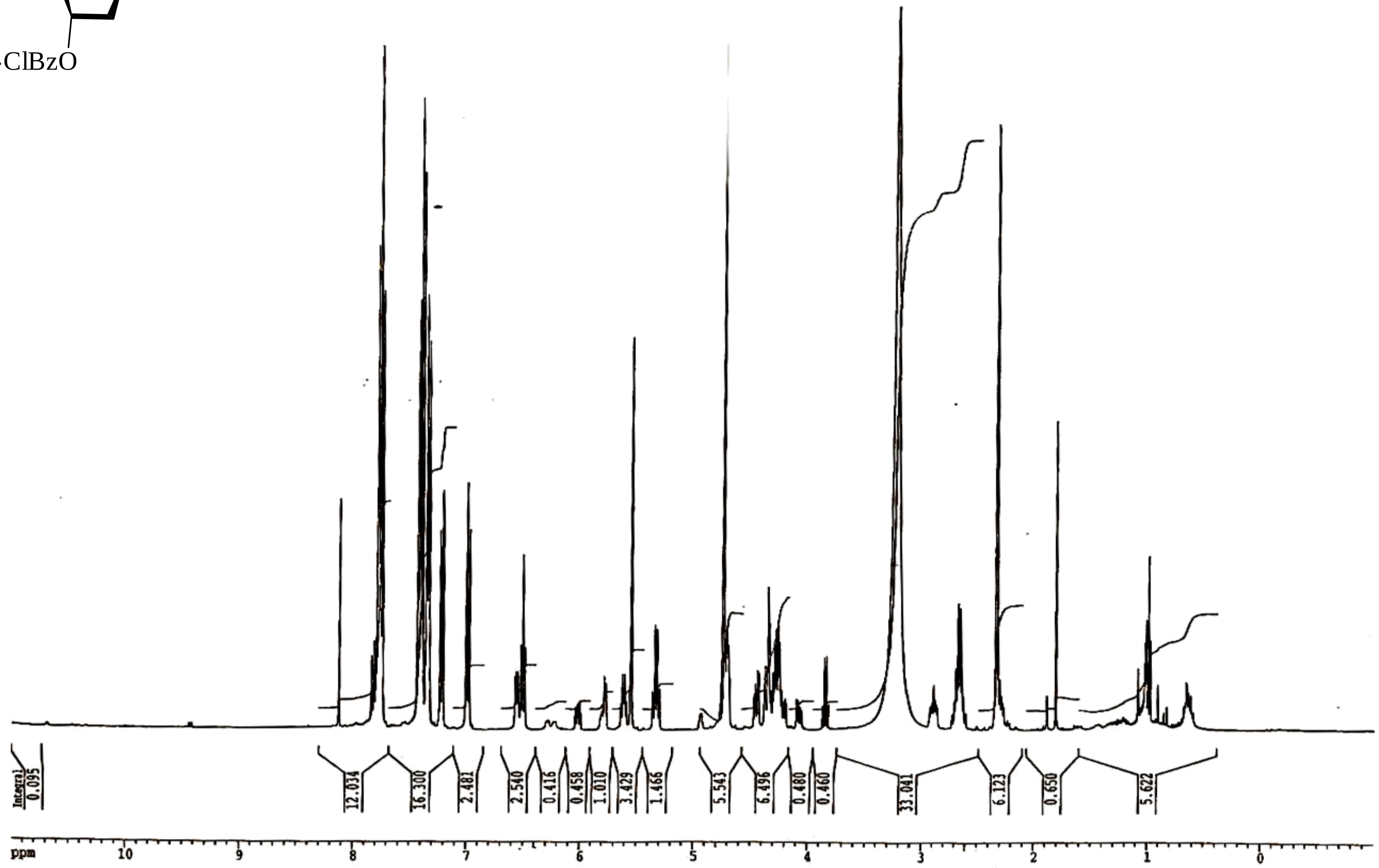

Current AMX400 Data	
DU	/x
NAME	20070703
EXPNO	220
PROCNO	1

F2 - Acquisition Parameters	
Date_	20070703
Time	23.05
INSTRUM	spect
PROBHD	5 mm Dual 13
PULPROG	zg30
TD	65536
SOLVENT	DMSO
NS	16
DS	2
SWH	8333.373 Hz
FIDRES	0.127157 Hz
AQ	3.9322100 sec
RG	256
DW	60.000 usec
DE	85.71 usec
TE	300.0 K
HL1	1 dB
D1	1.00000000 sec
P1	12.50 usec
SFO1	400.1387237 MHz
NUCLEUS	1H

F2 - Processing parameters	
SI	32768
SF	400.1363549 MHz
WDW	EM
SSB	0
LB	0.30 Hz
GB	0
PC	1.00

1D NMR plot parameters	
CX	30.00 cm
F1P	11.000 ppm
F1	4401.50 Hz
F2P	-1.000 ppm
F2	-400.14 Hz
PPMCM	0.40000 ppm/cm
HZCM	160.05453 Hz/cm

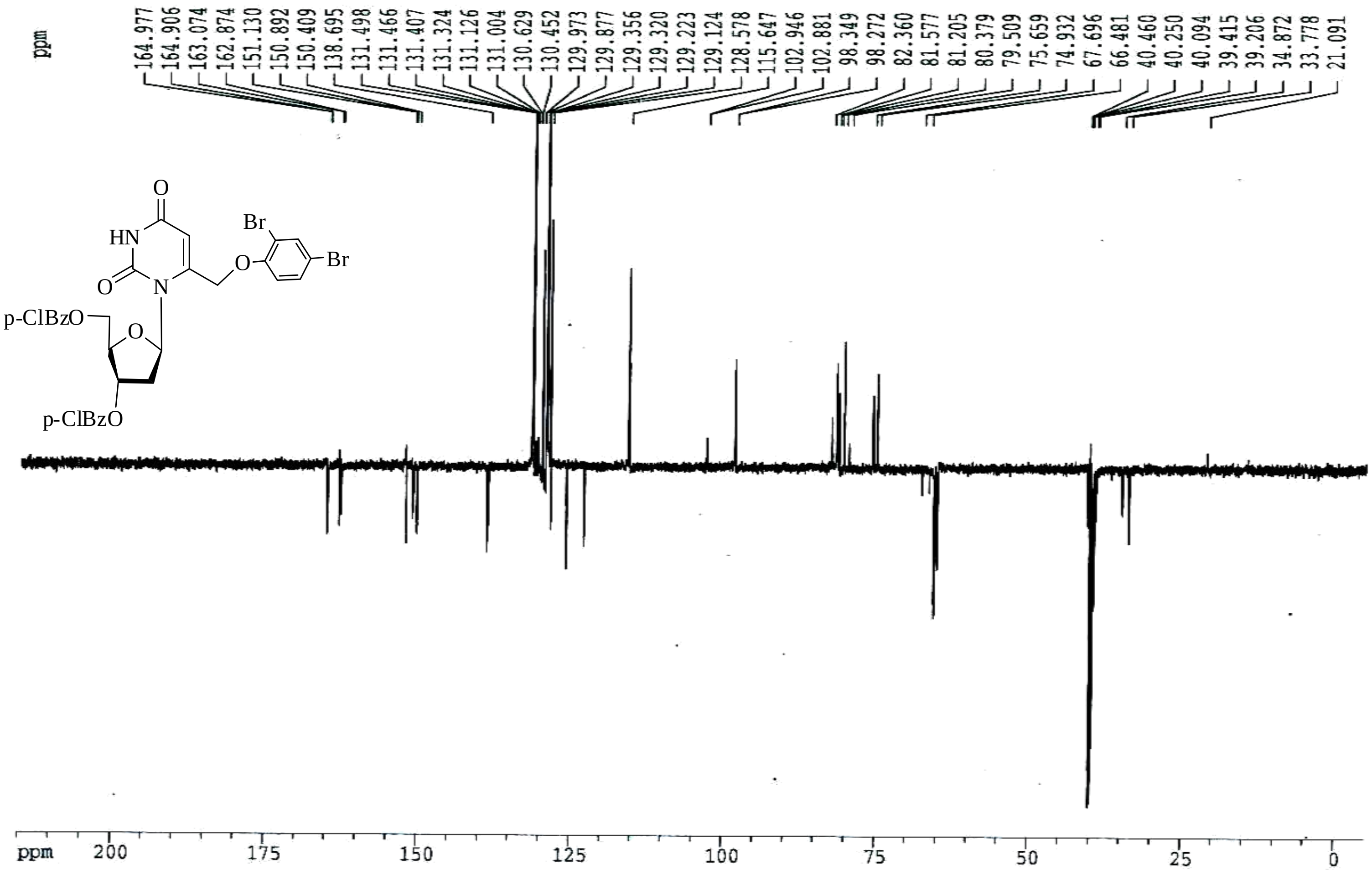

Current AMX400 Data
DU /x
NAME 20070709
EXPNO 50
PROCNO 1

F2 - Acquisition Parameters
Date_ 20070709
Time 17.32
INSTRUM spect
PROBHD 5 mm Dual 13
PULPROG pendant.amx
TD 65536
SOLVENT DMSO
NS 512
DS 4
SWH 25000.119 Hz
FIDRES 0.381472 Hz
AQ 1.3107700 sec
RG 32768
DW 20.000 usec
DE 28.57 usec
TE 300.0 K
P1 6.50 usec
P3 10.60 usec
CNST2 145.0000000
HL1 1 dB
D1 1.50000000 sec
S1 1 dB
SFO2 400.1379006 MHz
DECNUC 1H
D4 0.0017241 sec
P4 21.2 usec
P2 13.0 usec
D15 0.0043103 sec
D13 0.0000040 sec
S2 26 dB
SFO1 100.6245885 MHz
NUCLEUS 13C
CPDPRG waltz16
P31 90.00 usec
D2 0.00357143 sec

F2 - Processing parameters
SI 32768
SF 100.6143589 MHz
WDW EM
SSB 0
LB 1.00 Hz
GB 0
PC 1.40

1D NMR plot parameters
CX 20.00 cm
F1P 215.000 ppm
F1 21632.09 Hz
F2P -5.000 ppm
F2 -503.07 Hz
PPMCM 11.00000 ppm/cm
HZCM 1106.75793 Hz/cm

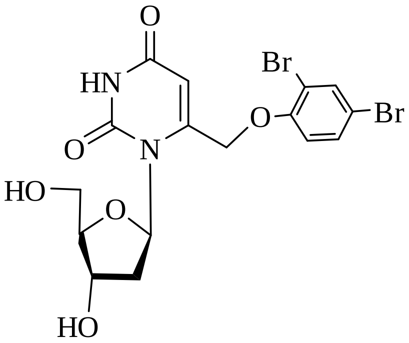

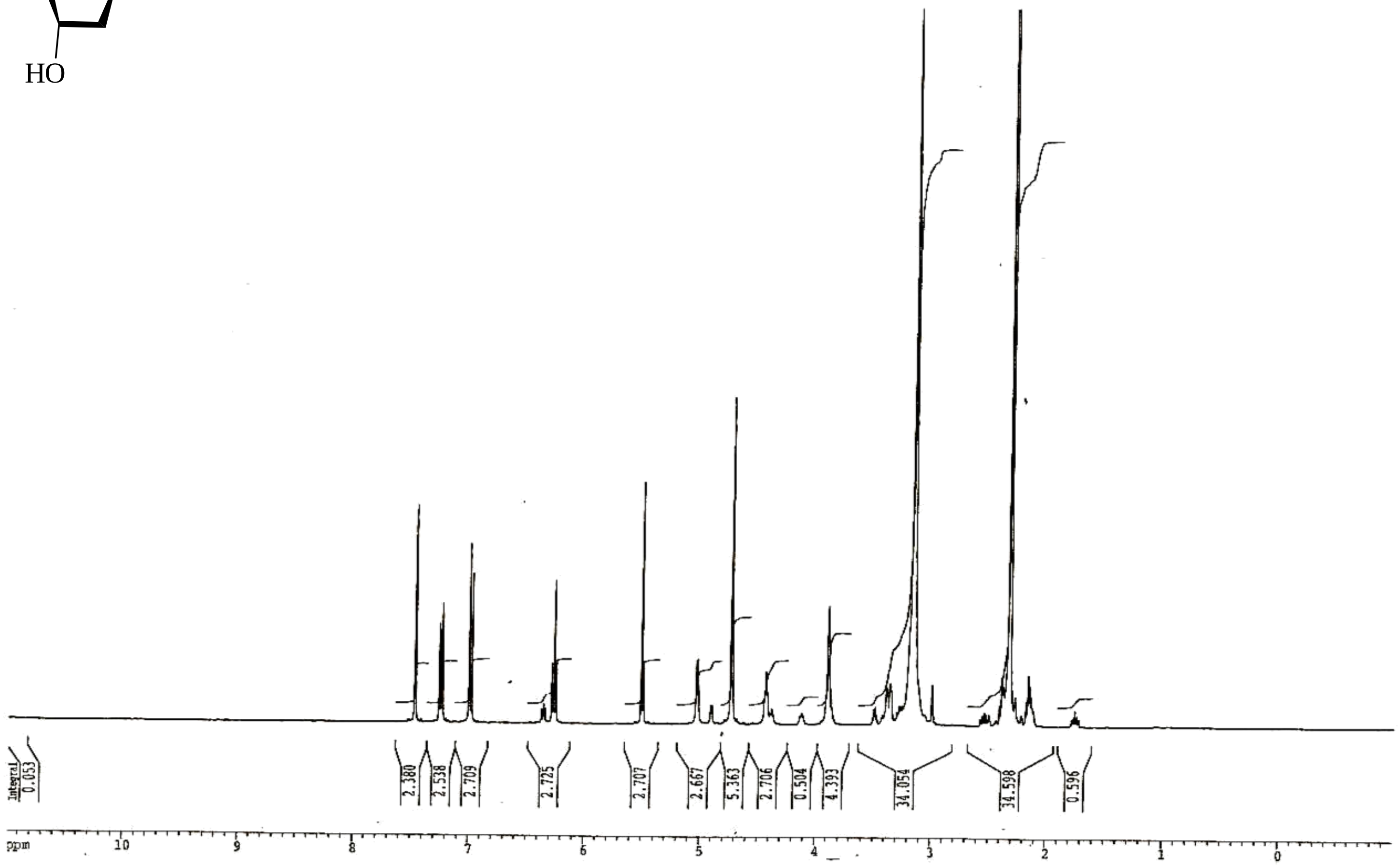

Current AMX400 Data
DU /x
NAME 20071004
EXPNO 280
PROCNO 1

F2 - Acquisition Parameters
Date_ 20071005
Time 6.02
INSTRUM spect
PROBHD 5 mm Dual 13
PULPROG zg30
TD 65536
SOLVENT DMSO
NS 16
DS 2
SWH 8333.373 Hz
FIDRES 0.127157 Hz
AQ 3.9322100 sec
RG 512
DW 60.000 usec
DE 85.71 usec
TE 300.0 K
HL1 1 dB
D1 1.00000000 sec
P1 12.50 usec
SFO1 400.1387237 MHz
NUCLEUS 1H

F2 - Processing parameters
SI 32768
SF 400.1363574 MHz
WDW EM
SSB 0
LB 0.30 Hz
GB 0
PC 1.00

1D NMR plot parameters
CX 30.00 cm
F1P 11.000 ppm
F1 4401.50 Hz
F2P -1.000 ppm
F2 -400.14 Hz
PPMCM 0.40000 ppm/cm
HZCM 160.05453 Hz/cm

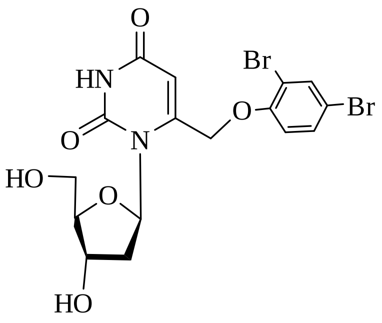

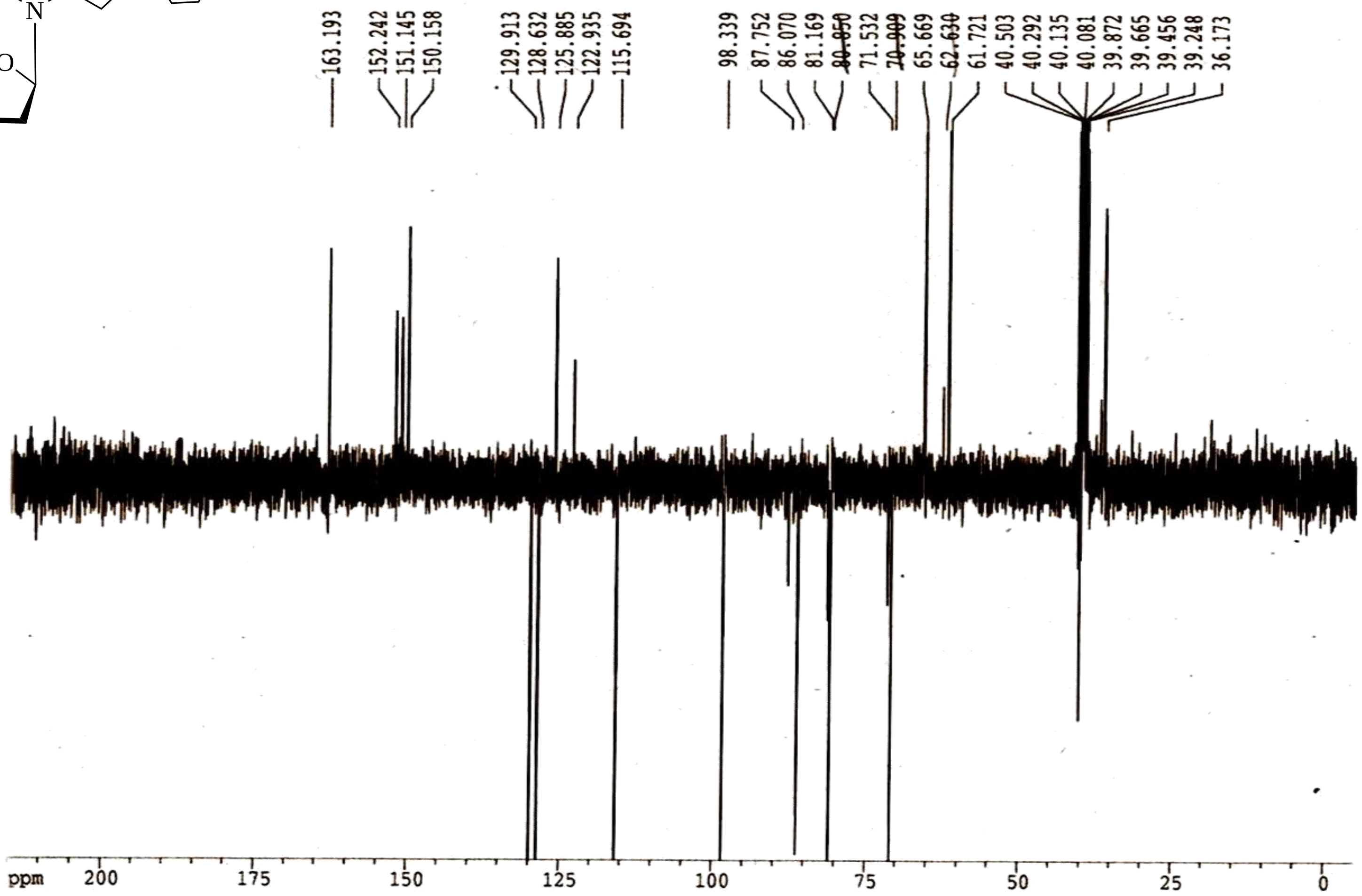

Current AMX400 Data

DU	/x
NAME	20071005
EXPNO	70
PROCNO	1

F2 - Acquisition Parameters

Date_	20071005
Time	15.19
INSTRUM	spect
PROBHD	5 mm Dual 13
PULPROG	pendant.amx
TD	65536
SOLVENT	DMSO
NS	800
DS	4
SWH	25000.119 Hz
FIDRES	0.381472 Hz
AQ	1.3107700 sec
RG	32768
DW	20.000 usec
DE	28.57 usec
TE	300.0 K
P1	6.50 usec
P3	10.60 usec
CNST2	145.0000000
HL1	1 dB
D1	1.50000000 sec
S1	1 dB
SFO2	400.1379006 MHz
DECNUC	1H
D4	0.0017241 sec
P4	21.2 usec
P2	13.0 usec
D15	0.0043103 sec
D13	0.0000040 sec
S2	26 dB
SFO1	100.6245885 MHz
NUCLEUS	13C
CPDPRG	waltz16
P31	90.00 usec
D2	0.00357143 sec

F2 - Processing parameters

SI	32768
SF	100.6143589 MHz
WDW	EM
SSB	0
LB	1.00 Hz
GB	0
PC	1.40

1D NMR plot parameters

CX	20.00 cm
F1P	215.000 ppm
F1	21632.09 Hz
F2P	-5.000 ppm
F2	-503.07 Hz
PPMCM	11.00000 ppm/c
HZCM	1106.75793 Hz/cn

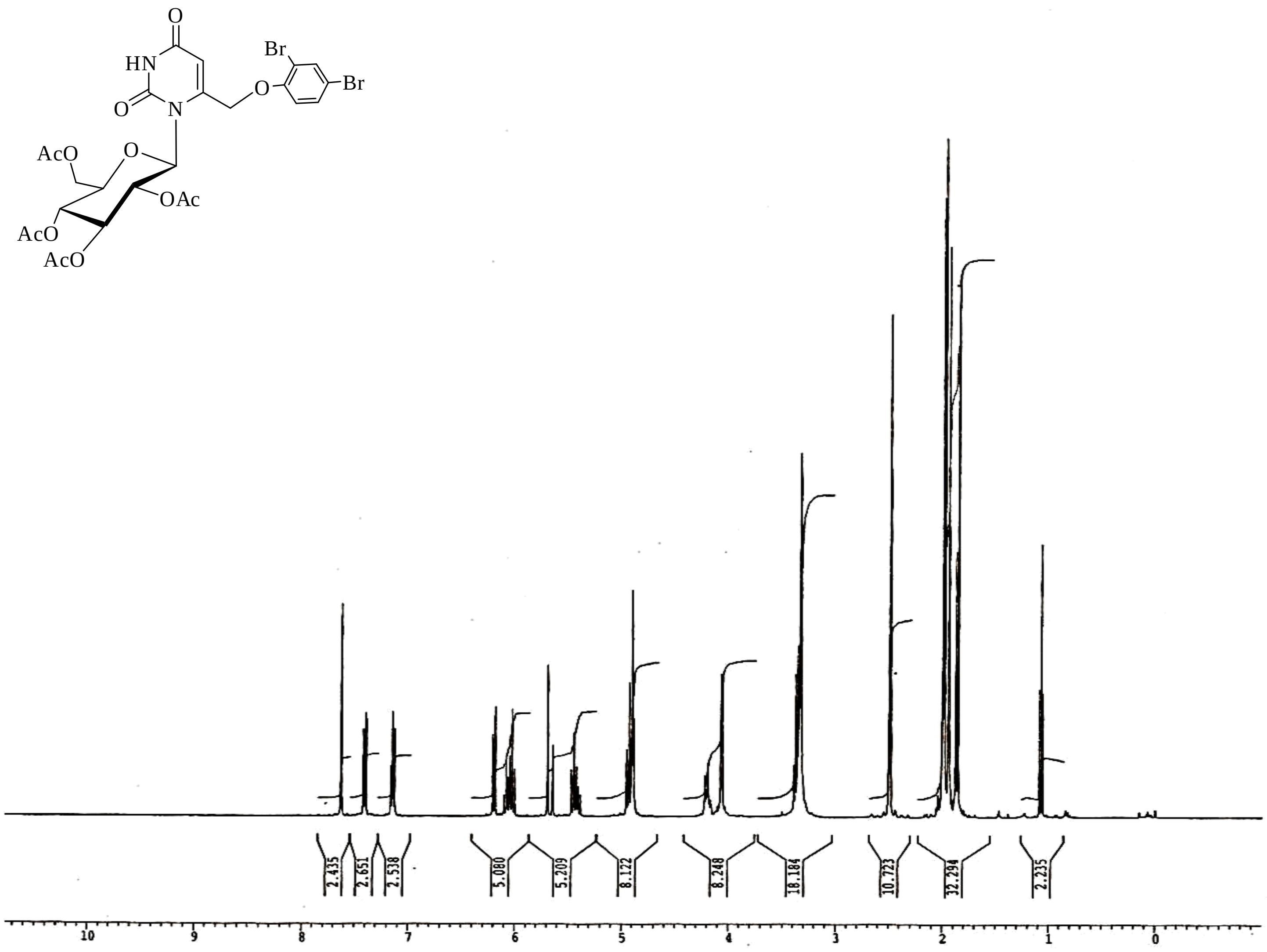


```
Current AMX400 Data
DU                      /x
NAME              20071004
EXPNO                  270
PROCNO                   1

F2 - Acquisition Parameters
Date_             20071005
Time                  5.51
INSTRUM              spect
PROBHD      5 mm Dual 13
PULPROG               zg30
TD                   65536
SOLVENT               DMSO
NS                      16
DS                       2
SWH              8333.373 Hz
FIDRES           0.127157 Hz
AQ              3.9322100 sec
RG                     256
DW                  60.000 usec
DE                   85.71 usec
TE                   300.0 K
HL1                      1 dB
D1              1.00000000 sec
P1                   12.50 usec
SFO1        400.1387237 MHz
NUCLEUS                 1H

F2 - Processing parameters
SI                   32768
SF          400.1362915 MHz
WDW                     EM
SSB                      0
LB                    0.30 Hz
GB                       0
PC                    1.00

1D NMR plot parameters
CX                   30.00 cm
F1P                 11.000 ppm
F1                 4401.50 Hz
F2P                 -1.000 ppm
F2                 -400.14 Hz
PPMCM              0.40000 ppm/cm
HZCM              160.05452 Hz/cm
```

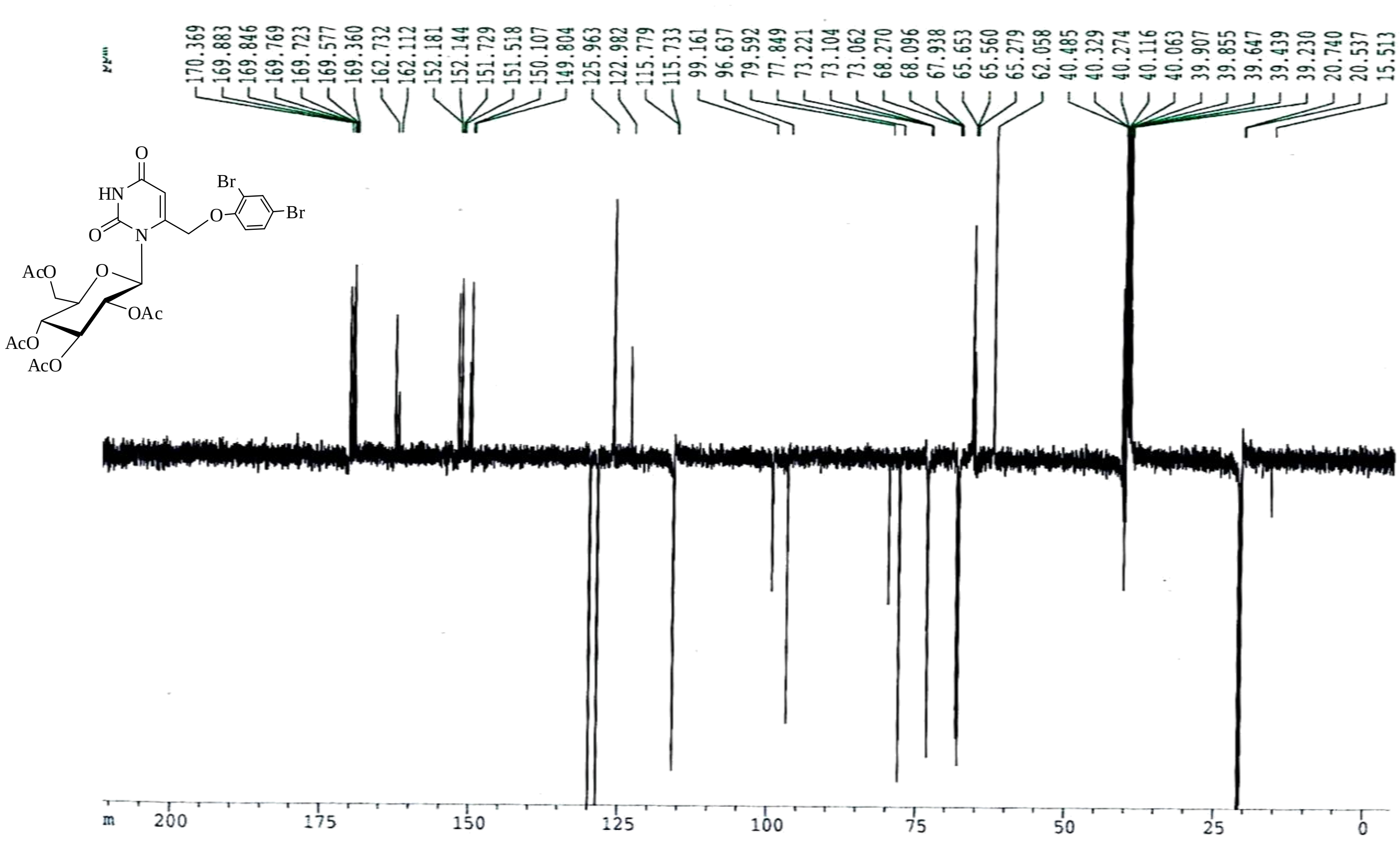


```
Current AMX400 Data
DU                      /x
NAME              20071005
EXPNO                   80
PROCNO                   1

F2 - Acquisition Parameters
Date_             20071005
Time                 16.06
INSTRUM              spect
PROBHD        5 mm Dual 13
PULPROG       pendant.amx
TD                   65536
SOLVENT               DMSO
NS                     800
DS                       4
SWH            25000.119 Hz
FIDRES          0.381472 Hz
AQ             1.3107700 sec
RG                   32768
DW                20.000 usec
DE                 28.57 usec
TE                 300.0 K
P1                  6.50 usec
P3                 10.60 usec
CNST2        145.0000000
HL1                    1 dB
D1            1.50000000 sec
S1                     1 dB
SFO2         400.1379006 MHz
DECNUC                  1H
D4             0.0017241 sec
P4                  21.2 usec
P2                  13.0 usec
D15            0.0043103 sec
D13            0.0000040 sec
S2                    26 dB
SFO1         100.6245885 MHz
NUCLEUS                13C
CPDPRG             waltz16
P31                90.00 usec
D2            0.00357143 sec

F2 - Processing parameters
SI                   32768
SF           100.6143589 MHz
WDW                     EM
SSB                      0
LB                    1.00 Hz
GB                       0
PC                    1.40

1D NMR plot parameters
CX                   20.00 cm
F1P                215.000 ppm
F1                21632.09 Hz
F2P                 -5.000 ppm
F2                 -503.07 Hz
PPMCM             11.00000 ppm/cm
HZCM            1106.75793 Hz/cm
```

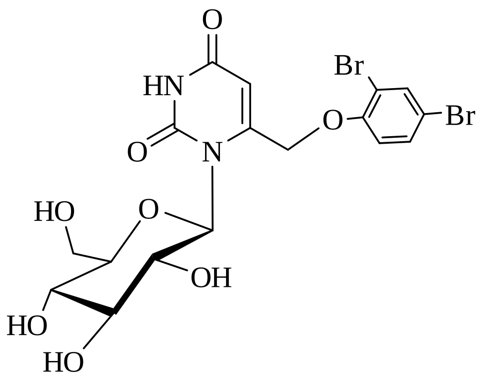

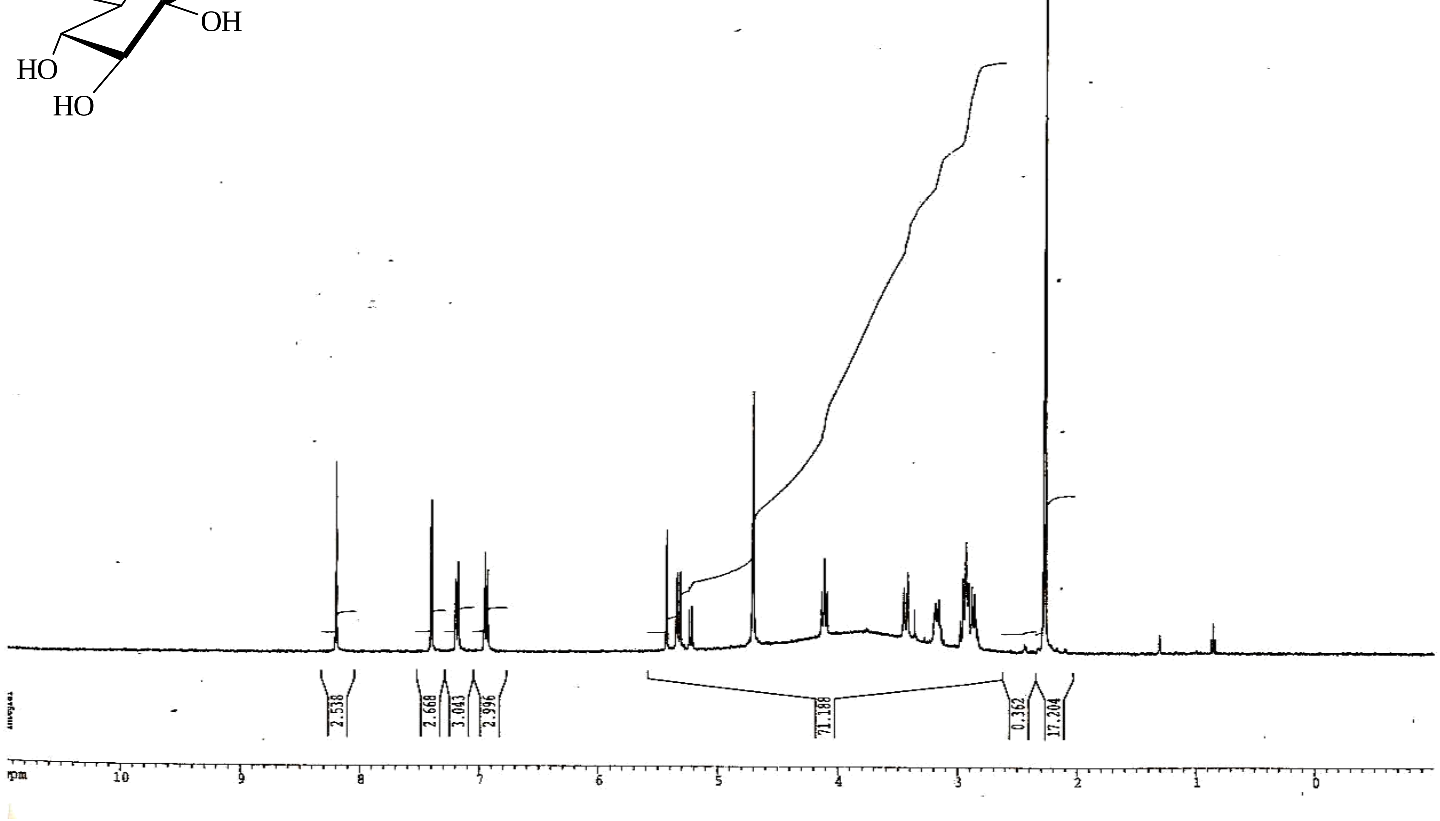


```
Current AMX400 Data
DU                      /x
NAME              20071102
EXPNO                  180
PROCNO                   1

F2 - Acquisition Parameters
Date_             20071102
Time                 17.15
INSTRUM              spect
PROBHD      5 mm Dual 13
PULPROG               zg30
TD                   65536
SOLVENT               DMSO
NS                      16
DS                       2
SWH               8333.373 Hz
FIDRES            0.127157 Hz
AQ               3.9322100 sec
RG                     512
DW                  60.000 usec
DE                   85.71 usec
TE                   300.0 K
HL1                      1 dB
D1              1.00000000 sec
P1                   12.50 usec
SFO1        400.1387237 MHz
NUCLEUS                 1H

F2 - Processing parameters
SI                   32768
SF          400.1363785 MHz
WDW                     EM
SSB                      0
LB                    0.30 Hz
GB                       0
PC                    1.00

1D NMR plot parameters
CX                   30.00 cm
F1P                 11.000 ppm
F1                 4401.50 Hz
F2P                 -1.000 ppm
F2                 -400.14 Hz
PPMCM              0.40000 ppm/cm
HZCM             160.05455 Hz/cm
```

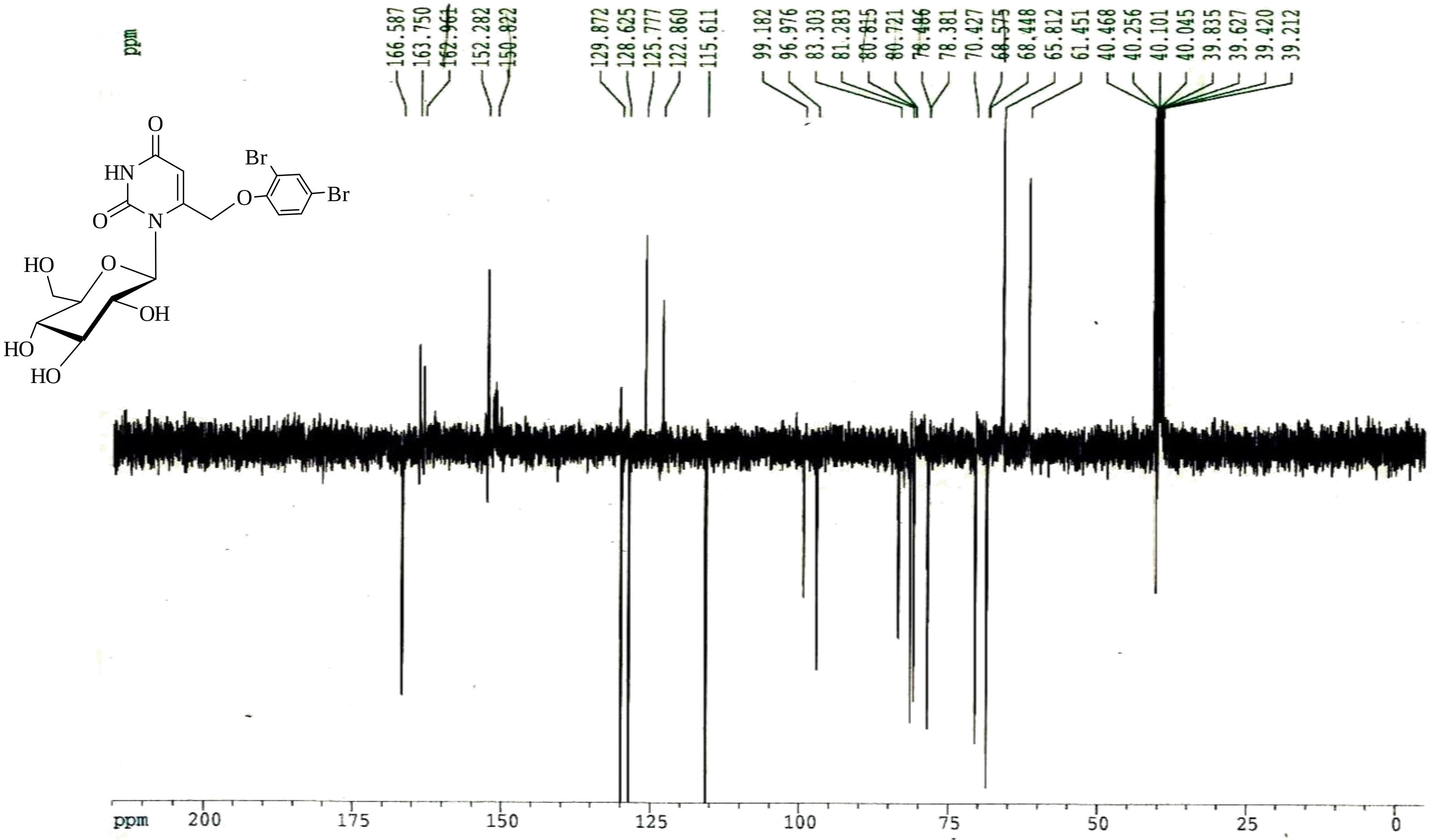

Current AMX400 Data
DU /x
NAME 20071105
EXPNO 260
PROCNO 1

F2 - Acquisition Parameters
Date_ 20071105
Time 21.03
INSTRUM spect
PROBHD 5 mm Dual 13
PULPROG pendant.amx
TD 65536
SOLVENT DMSO
NS 800
DS 4
SWH 25000.119 Hz
FIDRES 0.381472 Hz
AQ 1.3107700 sec
RG 32768
DW 20.000 usec
DE 26.57 usec
TE 300.0 K
P1 6.50 usec
P3 10.60 usec
CNST2 145.0000000
HL1 1 dB
D1 1.50000000 sec
S1 1 dB
SFO2 400.1379006 MHz
DECNUC 1H
D4 0.0017241 sec
P4 21.2 usec
P2 13.0 usec
D15 0.0043103 sec
D13 0.0000040 sec
S2 26 dB
SFO1 100.6245885 MHz
NUCLEUS 13C
CPDPRG waltz16
P31 90.00 usec
D2 0.00357143 sec

F2 - Processing parameters
SI 32768
SF 100.6143589 MHz
WDW EM
SSB 0
LB 1.00 Hz
GB 0
PC 1.40

1D NMR plot parameters
CX 20.00 cm
F1P 215.000 ppm
F1 21632.09 Hz
F2P -5.000 ppm
F2 -503.07 Hz
PPMCM 11.00000 ppm/cm
HZCM 1106.75793 Hz/cm
